# When learning with SLD is a hard challenge: a complex perspective on teacher-student relationships in primary school

**DOI:** 10.3389/fpsyg.2025.1595820

**Published:** 2025-10-09

**Authors:** Eleonora Florio, Alessia Cornaggia, Robert C. Pianta, Ilaria Castelli, Letizia Caso

**Affiliations:** ^1^Department of Human and Social Sciences, University of Bergamo, Bergamo, Italy; ^2^School of Education and Human Development, University of Virginia, Charlottesville, VA, United States; ^3^Department of Human Sciences, Libera Università Maria Ss. Assunta (LUMSA University), Rome, Italy

**Keywords:** primary school, teacher-student relationship, specific learning disorder, teacher attitudes, educational practices

## Abstract

**Introduction:**

The growing body of research highlighting the importance of a positive and supportive student-teacher relationship underscores the need to investigate whether pupils’ needs and teachers’ educational stance may shape the potential protective role of the student-teacher relationship. In line with our hypotheses, we investigated whether the presence of a Specific Learning Disorder (SLD) diagnosis, together with pupils’ and teachers’ demographic characteristics, may affect how both teachers and pupils perceive the quality of their relationship. We also examined teachers’ beliefs and attitudes toward SLD diagnoses and their educational stance, expecting that more adultcentric and authoritarian approaches may increase relational challenges when managing the complexity associated with SLD diagnoses.

**Methods:**

The present work addresses these aims, taking into account pupils’ and teachers’ perspectives on relational quality, teachers’ attitudes and beliefs regarding the rising number of SLD diagnoses in schools, and teachers’ agreement with the Adultcentric paradigm and Bleak Pedagogy. The study involved 66 Italian primary-school teachers and 110 Italian primary-school pupils, who completed validated questionnaires on student-teacher relationship quality, teachers’ attitudes toward SLD diagnoses, and educational stance. Data were analyzed through correlations, paired-sample *t*-tests, and linear mixed-effects models (LMMs) to examine associations between teachers’ beliefs, pupils’ reports, and perceptions of relational quality.

**Results:**

Results show that the student-teacher relationship may be partly influenced by the presence of an SLD diagnosis. The perceived levels of “Closeness” and “Dependency” were significantly higher when teachers described relationships with a pupil diagnosed with an SLD, compared to their descriptions of the relationship with an undiagnosed pupil with poor school performance.

**Discussion:**

Participants’ agreement with Adultcentrism and Bleak Pedagogy was associated with relationship quality and perceptions of SLD diagnosis, highlighting the substantial unsuitability of such educational stances with our evolving culture and society. Implications for intervention and future research are discussed.

## 1 Introduction

Over recent years, in Italy, within a general rise in neurodevelopmental disorders (Lucangeli and Perini, 2020), the number of students diagnosed with Specific Learning Disorder (APA, 2022) has notably increased. A report produced by the Statistical Office of the Ministry of Education and Merit (MIM, 2024) shows that the prevalence of Specific Learning Disorder (SLD) diagnoses rose from 0.9% in school year (SY) 2010/2011 to 6% in SY 2022/2023. Looking at the different types of SLD, the past decade has seen a steady increase across all categories: the number of pupils certified with dyslexia has more than doubled, while cases of dysgraphia, dysorthography, and dyscalculia have more than tripled. Considering only primary school, the percentage of pupils diagnosed with SLD increased from 0.8% in SY 2010/2011 to 3.2% in SY 2022/2023. This increase should also be considered in light of the fact that, starting in 2010 with the enactment of Law 170/2010, awareness of SLDs in Italy significantly grew, contributing to a broader recognition and reporting of such cases.

### 1.1 Rising SLD diagnoses and educational challenges

As a consequence of the increasing number of SLD diagnoses, the current expectation for teachers to manage a wide variety of special educational needs (e.g., Specific Learning Disorder - SLD) and to promote an inclusive classroom atmosphere (Cornoldi et al., 1998; Saloviita and Consegnati, 2019) requires them to continuously adjust and update their educational beliefs and methods to face the challenges of the current school context and the needs of individual pupils. The following section outlines the theoretical background and research context that motivate the present study, highlighting current educational challenges linked to the growing prevalence of SLD diagnoses, the potential effects of labeling, the importance of teacher-student relationships, and the role of educational stance, concluding with the aims and hypotheses of the study.

### 1.2 The protective role of teacher-student relationships

Given the challenges arising from the increasing complexity and broader acknowledgment of pupils’ varied needs, it becomes crucial to understand how the quality of the relationship between teachers and pupils can serve as a protective factor in promoting positive learning and developmental outcomes. In fact, the establishment of a supportive relationship between teacher and pupil is an important element of protection that is advantageous to the development of the child (Pianta, 2001a). The systematic review conducted by García-Rodríguez et al. (2023) highlighted that teachers’ attachment style and their availability in responding to student needs are associated with students’ school adjustment. The Student-Teacher Relationship Scale (Pianta, 1994, 2001b; Pianta et al., 1995; Pianta and Nimetz, 1991; Pianta and Steinberg, 1992), identified as “the most frequently used measurement instrument” (García-Rodríguez et al., 2023, p. 24), highlights various beneficial aspects of a relationship characterized by high levels of closeness and low levels of conflict and dependency within the theoretical framework of the attachment theory (Sabol and Pianta, 2012). Research consistently highlights the association of the quality of the teacher-student relationship with multiple components of pupils’ school experience, such as students’ engagement (Thornberg et al., 2022), self-efficacy in both the learning domain and academic success (Jederlund and von Rosen, 2023), concept-of-self, emotional regulation (García-Rodríguez et al., 2023), and cognitive abilities (Sankalaite et al., 2023; Semeraro et al., 2014). It is worth noting, however, that individual differences may also shape these relationships. From both teachers’ and pupils’ perspectives, some results in the literature on the quality of the pupil-teacher relationship suggest the presence of gender and age differences: females seem to build relationships characterized by greater closeness and less conflict (Koomen and Jellesma, 2015; Zee and Koomen, 2017; Zee et al., 2020). Therefore it is particularly important to highlight that, in the Italian context, these results could also be influenced by the prevalence of female teachers, especially in primary schools (La Grutta et al., 2023; Longobardi et al., 2016). Considering age differences, some studies showed a decrease in school attachment in the last years of primary school (Guidetti et al., 2017; Schneider et al., 2008; Zee et al., 2020). Consequently, it could be hypothesized that the overall quality of teacher-student relationships also declines during these final years, making age a relevant factor to consider among the other factors examined in this study.

Finally, previous studies suggest that higher workload and accumulated fatigue may hinder the ability to sustain consistent emotional attunement both with pupils in general and with those requiring more complex support (Bodenheimer and Shuster, 2020; Jennings and Greenberg, 2009; Maas et al., 2021). Therefore, among the factors that shape teachers’ perceptions, professional characteristics such as teaching experience and weekly working hours can be considered relevant, as they may influence the emotional resources and responsiveness available to build high-quality relationships with pupils.

### 1.3 SLD labeling and its impact on teachers’ perceptions

Beyond teachers’ professional characteristics, the quality of teacher-student relationships may vary significantly when pupils face learning challenges or carry diagnostic labels, such as those related to SLD, which can influence teachers’ perceptions and attitudes. Building on the literature showing the protective role of the teacher-student relationship, it is important to examine how this role applies when students face significant learning challenges, such as SLD. Pennings et al. (2016) emphasize the association between a high-quality teacher-student relationship and teachers’ ability to respond to their students’ needs. More specifically, Zee et al. (2020) studied the influence of the teacher-student relationship in a sample of primary school pupils who were diagnosed with dyslexia, an autism spectrum disorder (ASD), or attention deficit hyperactivity disorder (ADHD). They found that the impact of the relationship quality seemed to be higher in pupils with behavior-related disorders rather than in pupils with learning disorders (Zee et al., 2020). Nevertheless, the multi-informant approach also enabled the detection of some differences in the quality of the relationship as perceived by teachers, classmates, and pupils with a dyslexia diagnosis (Zee et al., 2020). Although all informants agreed on the lower level of conflict in the relationship, pupils with a dyslexia diagnosis, unlike both teachers and classmates, perceived less closeness with their teachers compared to pupils without this type of diagnosis (Zee et al., 2020). These differences in pupils’ and teachers’ perspectives suggest the relevance of collecting both teachers’ and pupils’ points of view, as done in the present study. Moreover, the perception of lower emotional availability of teachers coming from children with learning disorders in comparison with pupils without a diagnosis, detected also in previous studies (Al-Yagon and Mikulincer, 2004; Murray and Greenberg, 2000), provides a first reason to explore the quality of the teacher-student relationship in a sample of pupils an SLD diagnosis. The second reason stems from the investigation of teachers’ perspectives. In their relationship with pupils having a Specific Learning Disorder, teachers reported a lower perception of emotional closeness (Al-Yagon and Mikulincer, 2004) and a higher dependency in the relationship (Pasta et al., 2013) in comparison with the quality of the relationship with pupils without this diagnosis.

The understanding of teachers’ attitudes toward pupils with an SLD diagnosis has been explored in the literature using both implicit and explicit measures. The use of implicit measures detected the presence of a “labeling bias” of teachers toward pupils with dyslexia (Hornstra et al., 2010), suggesting that the diagnosis itself may influence teachers’ perceptions beyond the observable learning difficulties. Therefore, distinguishing between pupils with an SLD diagnosis and low achievers without this diagnosis is crucial to disentangle the effects of SLD labeling from those of performance.

At a more explicit level of investigation, studies explored educators’ perceptions (Tosun et al., 2021) and misconceptions concerning the knowledge of dyslexia (Soriano-Ferrer and Echegaray-Bengoa, 2014; Soriano-Ferrer et al., 2016; Wadlington and Wadlington, 2005). The role of primary school teachers’ knowledge about SLD was found to be a predictor for the use of compensatory tools, and teachers’ beliefs and emotions about SLD can predict the use of dispensatory accomodations (Re et al., 2021), showing the connection between teachers’ representational framework and their educational practice. Moreover, Tosun et al. (2021) revealed a weak correlation between teaching experience and the negative perception of dyslexia. The explorative study conducted by Cornoldi et al. (2018) extended the focus on primary school teachers’ attitudes toward learning disabilities, including beliefs about the roles of legislation, teachers, specialized intervention, compensatory measures, integration in the classroom, the labelling effect and emotional challenges in the experience of their pupils with learning disabilities. Although some beliefs were shared across the three countries involved in the study (Italy, Spain, and the U.S.), significant differences emerged (Cornoldi et al., 2018), suggesting the relevance of investigating the multiple variables that could intervene in influencing teachers’ attitudes and beliefs that guide their educational practices.

### 1.4 Educational stance and relationship quality in the classroom

In addition to diagnostic labels, another key factor to consider is the broader perspective of the educational stance that shapes teachers’ approaches to relationships with pupils and classroom management. To do so, it seems necessary to develop a deeper understanding of teachers’ ideas concerning an SLD diagnosis within a comprehensive and complex framework of beliefs and attitudes that contribute to defining the teacher’s educational stance (Florio, 2018). Adultcentrism and Bleak Pedagogy are two constructs that enable the exploration of specific beliefs and attitudes influencing teachers’ educational stance, as manifested in their methodological and classroom management choices. Indeed, as recognized by students, to define a “good teacher,” it is necessary to consider the interconnection among personal, relational, methodological, and management factors (Thornberg et al., 2022). Adultcentrism (Furioso, 2000; Goode, 1986) is an implicit paradigm of thought rooted in a sociocultural framework in which adulthood is regarded as the full realization of human development, in a dichotomous comparison with the perceived incompleteness of childhood (Florio et al., 2020a). Although adults intend to act in the best interests of children, the power asymmetry and the depiction of children as incomplete, immature, and incompetent individuals that characterized the adultcentric perspective could lead to inadequate recognition, understanding, and responses to children’s needs (Florio, 2018; Florio et al., 2020a; Mackay, 2003). Florio et al. (2022a) demonstrated that, in a sample of primary school teachers, an adultcentric perspective is a consistent predictor of the values and beliefs of an old-fashioned and authoritarian educational stance, namely the construct of Schwarze Pädagogik (Rutschky, 1977, 2015), which literally translates as Black Pedagogy (Florio et al., 2020b). It is necessary to clarify that this label does not pertain in any way to education involving black students and teachers, nor school programs including black studies (Johnson et al., 2014; Pitre et al., 2008): the label has been recently adapted, and the term currently used by this group of authors is Bleak Pedagogy (Florio et al., 2024a, 2024b; Florio et al., n.d.). Bleak Pedagogy comprises values, beliefs, and methods focused primarily on shaping the pupils according to ideal standards of educators and society, thereby undermining the pupils’ will (Florio et al., 2020b). The Bleak Pedagogy methods also include forms of both physical and psychological violence for disciplinary purposes (Florio et al., 2020b; Perticari, 2016). The sociocultural context also influences educators’ ability to recognize this detrimental educational stance, which – especially in its psychological forms – tends to remain implicit and, for this very reason, is often enacted in practice (Florio et al., 2022a). The first evidence (Florio, 2018) suggested that the agreement with Adultcentrism and especially Bleak Pedagogy could predict specific features of teachers’ representations concerning an SLD diagnosis, such as a critical attitude toward the role of society (e.g., medicalization, how children are raised nowadays), specialists, parents, and classmates in determining the presence and the school experience of pupils with an SLD diagnosis. These results underline the importance of investigating teachers’ attitudes and beliefs regarding their complex role and its impact on the quality of the teacher-student relationship, especially when dealing with a pupil diagnosed with an SLD or perceived as demonstrating low academic achievement.

### 1.5 From theory to research framework

Despite the growing body of research on teacher-student relationships and SLD, few studies have directly compared teachers’ perceptions of pupils with a formal SLD diagnosis and low achievers without a diagnosis. This comparison is particularly relevant for disentangling the potential effects of labeling from the underlying learning difficulties. Building on the literature on labeling bias (Hornstra et al., 2010) and regarding teachers’ attitudes toward SLD (Cornoldi et al., 2018; Tosun et al., 2021), our study aims to examine how teachers perceive and describe the quality of their relationships with these two groups of pupils. We also explore whether factors such as teaching experience and weekly working hours are associated with these perceptions, as suggested by previous findings on teacher fatigue and workload (Zee et al., 2020). Specifically, we ask whether the quality of teacher-student relationship differs between pupils with and without an SLD diagnosis, and how teachers’ professional characteristics—such as educational stance—might influence their views. These considerations form the basis of our study objectives and hypotheses, outlined below. Building on these premises, the present study integrates variables such as labeling effects, teacher workload, experience, and pupils’ age, to offer a multi-layered perspective that extends existing research on teacher-student relationships and SLD.

### 1.6 Aims of the study

The present study explores the impact of teachers’ attitudes and beliefs regarding SLD diagnosis and their educational role in the quality of the teacher-pupil relationship, investigated from the perspectives of both the teachers and their pupils seen as active constructors of the relationship.

The first objective is to investigate perceptions of the quality of the teacher-student relationship with pupils who have an SLD diagnosis (Specific Learning Disorder diagnosis—SLD pupils). The first hypothesis (A) is that the presence of the diagnostic label of a Specific Learning Disorder could influence teachers’ and pupils’ representations of their relationship, even when compared with pupils who, in the eyes of teachers, have some difficulties in dealing with learning challenges (Without a Diagnosis - WD pupils) (Hornstra et al., 2010; Pasta et al., 2013; Re et al., 2021; Zee et al., 2020). The second hypothesis (B) is that pupils’ age and gender, as well as teachers’ experience and working hours, may influence the quality of the teacher-student relationship, as highlighted by previous literature (Guidetti et al., 2017; La Grutta et al., 2023; Longobardi et al., 2016; Tosun et al., 2021).

The second objective is to enrich the perspective on the quality of student-teacher relationship through the investigation of teachers’ beliefs and attitudes toward SLD diagnosis and the paradigm behind their educational approach, considering also the possible influence of teachers’ demographics. The first hypothesis (C) is that adult-centered and authoritarian educational practices are associated with difficulties in the educational relationship and greater teacher fatigue in dealing with the complexity that SLD diagnoses bring to the table. The second hypothesis (D) is that teachers’ beliefs and attitudes toward learning disabilities could influence both the pupils’ school experience (Re et al., 2021; Zee et al., 2020) and how teachers perceive the relational quality with pupils (Pasta et al., 2013).

## 2 Materials and methods

### 2.1 Participants and procedures

Teachers and children belonged to 26 different primary schools located in the province of Bergamo, in Italy. All participants were treated in accordance with the Declaration of Helsinki (World Medical Association, 2013), as well as with the ethical guidelines for research provided by the American Psychological Association (APA, 2017) and the Italian Psychological Association (AIP, 2022). Participants were also provided with the researcher’s contact information to receive further clarifications if needed, and informed consent was obtained to proceed with the battery of questionnaires. Participants received clear information about the objectives and phases of the research and about their rights as participants, including the guarantee of anonymity and the possibility of dropping out of the study at any moment. The following subsections describe the characteristics of the two participating groups—teachers and pupils—as well as the procedures adopted for each group.

#### 2.1.1 Teachers

The age range of the teacher sample (*N* = 66) was 27 to 63 years (*M* = 48, *SD* = 7.6). The average number of years of teaching experience was 21.7 (*SD* = 11.4), whereas the average number of teaching hours per week was 21.2 (*SD* = 3.5). Teachers employed in a curricular role represented 90.9% of the sample, while 9.1% were special needs teaching assistants. The sample included only female teachers; 75.8% of the sample had an upper secondary school qualification, 9.1% a “University Diploma” (qualification established by Law 341/90, no longer in force), 1.5% a bachelor’s degree, 10.6% a master’s degree and, lastly, 3% held a post-master’s specialization.

All measures described later and dedicated to the teachers were administered online by providing the link to an online survey platform. Matching the teacher’s and the student’s answers was possible because STRS required teachers to explicitly state the name of the pupil they were thinking of while completing the questionnaire. During the data processing phase, a unique anonymous code was assigned to both the teachers and the pupils. This allowed us to link the answers of each teacher to the answers of the considered child, thus constructing an anonymous dataset for the analyses. It is of utmost importance to specify that a similar instruction was not part of the measurement tools administered to the children, i.e., they completed the Student-Teacher Relationship Questionnaire (STRQ) thinking of their group of teachers in general—necessarily including the teacher who had completed the Student-Teacher Relationship Scale (STRS). This aspect will be further discussed in the limitation section.

#### 2.1.2 Pupils

The sample of children included 110 primary school pupils from 2nd to 5^th^ grades (65% males, 35% females), with an age range from 7 to 12 years old (*M* = 9.1, *SD* = 1.2). The continuous age scale was divided into two categories to allow age-group comparisons: 55.5% “younger” children (*M* = 8.2 y, *SD* = 0.7) attending 2nd and 3rd grades, and 44.5% “older” children (*M* = 10.1 y, *SD* = 0.7) attending 4th and 5th grades. Teachers were asked to indicate two pupils in their classes: one child with an SLD diagnosis (hereafter “SLD pupils”), and one child without a diagnosis but with poor academic performance (hereafter “WD pupils”). This produced two subsamples of 55 children each. The first subsample consisted of SLD pupils aged 7 to 12 (*M* = 9.2, *SD* = 1.2), 68% were male, and 52.7% were “younger” children. The group of children evaluated by teachers as pupils with poor school performance but without an SLD diagnosis included children aged 7 to 11 (*M* = 8.9, *SD* = 1.2), 60% of them were male, and 58.2% fell into the “younger” category.

Parents gave their informed consent for children’s participation by completing a form that was made available for them both online and on paper. On the same occasion, parents were also asked to provide some information about their participating child (e.g., age, gender, presence of SLD diagnosis, etc.). The administration of the instrument to children took place in person, in their own schools and in group sessions of about 15–20 children each, conducted by two or three researchers. To start, children were given a clear and simple explanation of how the activity would take place. Following this introduction, one researcher read the questions aloud, leaving the children time to mark their answers and ask questions while completing the initial fields (age, gender, favorite subject, etc.) and STRQ. It is important to reiterate that, unlike teachers, children were not asked to focus on one specific teacher when completing the STRQ. They responded with reference to their teachers in general; therefore, the teacher who completed the STRS was necessarily included in the group they were referring to.

### 2.2 Measures

According to the aim of the study, measures investigating the quality of the teacher-student relationship were used. Both teachers’ perspectives (STRS - Student-Teacher Relationship Scale; Pianta, 1994, 2001b; Pianta et al., 1995; Pianta and Nimetz, 1991; Pianta and Steinberg, 1992) and pupils’ perspectives (STRQ - Student-Teacher Relationship Questionnaire; Murray and Greenberg, 2000; Tonci et al., 2012) were taken into account, and a measure to collect teachers’ attitudes and beliefs regarding SLD diagnosis (RADSA - Representations and Attitudes towards SLD diagnoses questionnaire; Florio et al., 2022b) was also included. In order to fulfill the second specific objective, the Bleak Pedagogy Scale (Florio et al., 2020b) and the Adultcentrism Scale for Teachers (Florio et al., 2022a) were used, allowing further exploration of the complexity of the educational relationship, including the paradigm of thought, values, and methods underlying teachers’ educational approaches.

#### 2.2.1 Measures administered to teachers

Teachers were asked to indicate one pupil with an SLD diagnosis (SLD pupils), and one pupil they considered to have poor school performance but without any diagnosis (WD pupils). In particular, to investigate the quality of the teacher-pupil relationship they were asked to complete the Italian version of the Student-Teacher Relationship Scale (Pianta, 1994, 2001b; Pianta et al., 1995; Pianta and Nimetz, 1991; Pianta and Steinberg, 1992) twice. The STRS is composed of 22 items in its Italian version validated by Fraire et al. (2013), reporting good internal consistency for the subscales (“Closeness”: *α* = 0.85; “Conflict”: *α* = 0.92; “Dependency”: *α* = 0.69). The initial instructions of the STRS ask teachers to think of a particular pupil while answering the questions. Subsequently, items describing relationship characteristics are presented, and participants indicate how much each statement reflects their relationship with the pupil in question. Responses are set on a 5-point Likert scale of applicability (1 = Definitely does not apply; 2 = Does not really apply; 3 = Neutral, not sure; 4 = Somewhat applies; 5 = Definitely applies). The Italian version of STRS maintains the original three-dimensional structure, composed of “Closeness” (8 items), “Conflict” (10 items) and “Dependency” (4 items). The “Closeness” subscale provides information about the degree of warmth, affection and open communication experienced by the teacher with a particular student; the “Conflict” subscale provides an evaluation of the level of negativity and conflict present in the relationship; lastly, the “Dependency” subscale measures the extent to which the teacher perceives the student as overly dependent (Pianta, 2001b). A higher total score on the STRS indicates an overall positive relationship, which tends to be characterized by lower levels of conflict and dependency, and by higher levels of closeness (Pianta, 2001b, p. 12).

##### 2.2.1.1 Teachers’ attitudes towards SLD diagnoses

In addressing the complexities associated with SLD diagnoses, the Representations and Attitudes towards SLD diagnoses questionnaire was chosen (RADSA; Florio et al., 2022b) as the tool that best allows teachers to examine their own representations. This makes evident possible implicit obstacles that may affect the quality of the relationship, especially with SLD pupils. The RADSA questionnaire, validated on a sample of Italian primary school teachers (Florio et al., 2022b), is composed of 62 items with a 4-point Likert scale measuring the level of agreement (1 = Fully disagree; 2 = Slightly agree; 3 = Agree; 4 = Fully agree). RADSA is organized in four sections: “Increase of SLD diagnoses,” “Peer group,” “Parents’ attitudes towards SLD diagnosis,” “The moment when the teacher refers parents to an SLD specialist.” Each section is composed of three factors. Given the multidimensional structure of the instrument, Table 1 illustrates its sections and factors, along with example items. For a comprehensive overview of the RADSA and its characteristics, (see Florio et al., 2022b). As no internal consistency estimates were reported in the original validation of the RADSA, we computed Cronbach’s *α* on the full set of items. The resulting internal consistency was good (*α* = 0.84).

**Table 1:** Representations and attitudes towards SLD diagnosis questionnaire (RADSA).

Sections and factors	Description	Item example
Section 1: diagnoses increase		
Medicalization (7 items)	high scores: medicalization tendency by professionals and society produces more SLD cases	The number of SLD diagnoses has increased due to the growing tendency in society to medicalize circumstances, which is becoming increasingly prevalent.
System-level causes (7 items)	high scores: today’s society produces more SLD cases	The contemporary parent’s lifestyle is characterized by a greater degree of frenetic activity.
How children are raised nowadays (4 items)	high scores: current child-rearing culture causes more SLD cases	The prevalence of SLD diagnoses has increased as a consequence of the pervasive use of technology (e.g., smartphones, tablets) which results in a greater number of intense and frequent stimuli for children in the present era.
Section 2: peer group		
Complaints about facilitations (6 items)	high scores: classmates usually complain about the facilitations granted to pupils with an SLD diagnosis	Other pupils complain in order to get the same facilitations as a classmate with SLD.
Attention to emotions and needs (6 items)	high scores: inclusive class climate is promoted by paying attention to emotions and individual needs; low scores: such attention is not necessary, since an inclusion climate occurs spontaneously	Creating an inclusive class climate means explaining to children that everyone has different needs.
Fairness of evaluation (5 items)	high scores: it is fair to differentiate tests and homework between pupils with/without an SLD	It is not reasonable to assign identical homework tasks to all pupils. It is necessary to provide each pupil with homework that is appropriate to their individual needs.
Section 3: parents’ attitudes towards SLD diagnosis		
Roles and information (5 items)	high scores: parents need more clarity on professional figures’ roles and on the nature of SLDs	Society should change its mindset and provide parents with training that is not “dropped from the top” so that they can really understand SLDs.
Diagnosis as alibi (4 items)	high scores: parents tend to attribute all problems to the SLD diagnosis	Parents use the diagnosis of an SLD as an “alibi”: they do not accept that teachers try to stimulate or encourage the pupil anyway.
Parents’ negative reactions to SLD diagnosis (3 items)	high scores: parents tend to react negatively to the idea that their child could have an SLD	Parents experience the diagnosis of an SLD as a wound.
Section 4: the moment in which the teacher refers parents to an SLD specialist		
Diagnosis usefulness (3 items)	high scores: the diagnosis is not particularly useful to teachers when dealing with a pupil with an SLD	The diagnosis of an SLD does not give the teacher any particular indication beyond what the teacher was already implementing to help the pupil.
Teacher positioning (7 items)	high scores: the teacher’s role is penalized in comparison to other professionals – e.g., neuropsychiatrists, psychologists, etc.	More frequent collaboration with a specialist would be needed in regard to each individual case as the worlds of teachers and specialists are currently separated.
Strengths and weaknesses (7 items)	high scores: strength and resourcefulness when working with a pupil with SLD	It is necessary to stimulate and encourage even pupils who have a diagnosis of SLD because they can still improve.

##### 2.2.1.2 Teachers’ educational stance

Teachers’ agreement with attitudes and methods typical of an old-fashioned and authoritarian educational stance was evaluated with the first section of the Bleak Pedagogy Scale (originally published as the Black Pedagogy Scale; Florio et al., 2020b). The first section is named “Bleak Pedagogy Observation” section (BPO), which consists of 24 items, with a Cronbach’s *α* of 0.87. Teachers are requested to express their degree of agreement with each item on a 4-point Likert scale. This section of the instrument is composed of three factors: “Values of Bleak Pedagogy” (*α* = 0.87; high values: agreement with the main educational values and objectives typical of the Bleak Pedagogy’s perspective), “Education of children over time” (*α* = 0.75; high values: nostalgic attitude towards old-fashioned educational practices as more effective and useful), and “Methods of Bleak Pedagogy” (*α* = 0.74; high values: agreement with Bleak Pedagogy disciplinary and educational methods, such as pedagogical beating, humiliation, coldness towards the child, etc.). The original instrument also includes a first item for temporal placement and a further a section assessing the perceived prevalence of Bleak Pedagogy methods in the past and today, which were not used in the present study. The Bleak Pedagogy Scale has been validated on a sample of Italian teachers (Florio et al., 2022a), following a first study (Florio et al., 2020b) conducted with a sample of Italian university students and parents.

The second measurement tool used is the Adultcentrism Scale for Teachers (ADT; Florio et al., 2022a), composed of 9 items (Cronbach’s *α* = 0.74) with a 4-point Likert scale based on agreement. High scores indicate that teachers tend to think of their pupils as disempowered and without responsibility, as an empty receptacle in need of being filled by adults with social and cultural values. The validation of this version of the instrument on the Italian teachers’ sample was conducted by Florio et al. (2022a) after the first validation study for the original scale (Florio et al., 2020a) with a sample of Italian parents and university students.

#### 2.2.2 Measure administered to pupils

Students play an active role in constructing the teacher-student relationship and determining its quality and dynamics (Pianta, 2001a). Therefore, to avoid a partial representation of this relationship, the Student-Teacher Relationship Questionnaire (Murray and Greenberg, 2000) in its Italian version (Tonci et al., 2012) was presented to children, to collect data on how they experience their relationship with their teacher. The instrument, validated by Tonci et al. (2012) on a sample of 4th and 5th grade Italian pupils, consists of 22 items with possible answers based on a 4-point Likert scale (1 = Not at all true; 2 = Not very true; 3 = Quite true; 4 = Very true), loading on the following three subscales: “Closeness with teachers” (*α* = 0.86; high scores: positive evaluation of the relationship in terms of emotions, closeness and support), “Dissatisfaction with teacher” (*α* = 0.76; high scores: child’s negative opinions and feelings concerning the teacher), and “Bond with school” (*α* = 0.71; high scores: positive feelings towards the school environment and activities). In our sample, Cronbach’s *α* exceeded 0.76 for the younger group and 0.74 for the older group. The coefficients, ranging from 0.742 to 0.875, are generally considered acceptable to good (DeVellis, 2016; George and Mallery, 2003; Taber, 2018). For each STRQ subscale, mean corrected item-total correlations (*r.drop*) were also computed: coefficients ranged from 0.481 to 0.682 across age groups. These values are considered acceptable and ideal values (Nunnally and Bernstein, 1994; Hair et al., 2019), supporting internal consistency alongside Cronbach’s *α*.

As described in the procedure section, in order to facilitate comprehension even by pupils in the lower grades as compared with the validation sample, all items were read aloud, with the researcher giving pupils the opportunity to ask for clarification. No critical issues were found in the completion of the work even by second and third graders.

### 2.3 Data analysis

The analyses were performed in R (Version 2025.05.0 + 496). Analyses were designed to address the specific hypotheses outlined in section 1.6 (Aims of the Study). The corresponding statistical procedures are briefly recalled below to guide the reader through the structure of the results. The absence of important violations of normality was assessed, as values of skewness and kurtosis between −1 and +1 are considered acceptable (Muthén and Kaplan, 1985). Thresholds of *sk* > 2 and *ku* > 7, values that would indicate a severe violation of normality, were not exceeded (Costello and Osborne, 2005; Kim, 2013; West et al., 1995). Regarding STRQ, internal consistency coefficients (Cronbach’s *α*) were computed separately for younger and older children, since the original validation of STRQ was conducted on 4th and 5th grade pupils only (Tonci et al., 2012). Values of *α* > 0.7 were considered acceptable, whereas *α* > 0.8 indicated good reliability (DeVellis, 2016; George and Mallery, 2003; Taber, 2018). We acknowledge that Cronbach’s *α* by itself provides only a partial index of scale quality and should not be interpreted as definitive evidence of dimensionality or construct validity; further analyses (e.g., item-total correlations or factor analysis) would be warranted to fully support the internal structure of the subscales (Taber, 2018).

However, we chose not to conduct factor analysis for several reasons: first, STRQ was used in its validated structure without modification. Second, as detailed communalities (*h^2^*) were not reported in the original Italian validation of STRQ (Tonci et al., 2012), it is difficult to assess whether our sample size meets the conditions for stable factor recovery: according to Hogarty et al. (2005), when *h^2^* are unknown or potentially low, sample to variable ratios (*N/p*) below 10:1—such as in our case (*N/p* = 6.5:1)—may be insufficient to ensure stability. Lastly, factor analysis was beyond the scope of the present study, which did not aim to revalidate STRQ on a younger sample. Instead, item-total correlations were computed using the “sirt” package (Robitzsch, 2024). The mean corrected item-total correlation (*r.drop*) is reported and interpreted following Nunnally and Bernsteof (1994), who suggest accepting values of *r.drop* ≥ 0.3, with values above 0.5 considered ideal (Hair et al., 2019).

To fulfill the first purpose of the study and to verify the related hypotheses (A and B), analyses considering teachers’ and pupils’ perceptions of the relationship involving SLD vs. WD students were carried out. These included descriptive statistics for STRS and STRQ subscales, correlations to detect associations between STRS subscales and STRQ scores, and paired *t*-tests to compare STRS and STRQ subscales scores. For paired-sample *t*-tests, Cohen’s *d* was calculated to estimate the magnitude of differences, in accordance with standard guidelines (Cohen, 1988). Correlations with teachers’ demographics and independent-sample *t*-tests have been conducted to detect significant differences in all variables based on each student’s school grade (i.e., younger: 2nd and 3rd grade, older: 4th and 5th grade) and gender, to verify the second hypothesis related to this first objective.

In line with the second aim of the study (hypotheses B and C), descriptive statistics for the other measures (i.e., RADSA and Adultcentrism/Bleak Pedagogy) were calculated. To verify the related hypotheses, correlations were performed among all the subscales and teachers’ demographics. Regarding Pearson product–moment correlations, Cohen’s guidelines (Cohen, 1988) were used to interpret their strength: values of *r* falling within the range of 0.50 to 1 are considered “large,” those between 0.30 and 0.49 “medium,” and those between 0.10 and 0.29 “small.” When significant correlations warranted further investigation, to account for the nested structure of the data (i.e., students nested within teachers and schools), Linear Mixed-effects Models (LMMs) were performed using the “lme4” package (Bates et al., 2015) for LMMs and the “lmerTest” package (Kuznetsova et al., 2017) to compute *p*-values via Satterthwaite’s approximation for degrees of freedom. When it was not possible to include both school and teacher as random intercepts due to convergence issues, only the school-level intercept was retained to account for the nested data structure. For these analyses, data were first restructured from wide to long format to allow for within-teacher comparisons, since each teacher completed STRS twice (once for a student with SLD and once for a student without a diagnosis). LMMs were also based on the relevant hypotheses: fixed effects included diagnostic status (SLD vs. WD), pupil demographics (gender, age group), teacher characteristics (experience, weekly hours), and theoretical variables such as Adultcentrism and Bleak Pedagogy scores. Random intercepts for school were included in all models, and random intercepts for teacher were added when multiple ratings per teacher were available. When teacher-level variance was negligible (singular fit), only school-level random intercepts were retained.

Intraclass correlation coefficients (*ICC*) from null models were computed to justify the multilevel approach and are presented in the Results section. *ICC*s were derived from the variance components obtained via the VarCorr{lme4} function (Devine et al., 2024). Values were interpreted as follows: *ICC* < 0.05 = poor, 0.05–0.75 = small to moderate, 0.75–0.90 = good, > 0.90 = excellent (Liljequist et al., 2019; Trevethan, 2017). However, it should also be noted that *ICC* values between 0.05 and 0.25 are quite common in social sciences (Snijders and Bosker, 2012). When the *ICC* was equal to 0, indicating no variance attributable to the grouping factor, the LMM approach was nonetheless retained if the model was statistically significant, to ensure consistency across analyses. To complement the information provided on each LMM, we computed marginal and conditional *R*^2^ values with “performance” package (Lüdecke et al., 2021). These quantify, respectively, the proportion of variance explained by the fixed effects alone (*R*^2^_m_) – providing insight into the effect size attributable to the predictors – and the total variance explained by the full model (*R*^2^_c_), accounting for both fixed and random effects (Johnson, 2014; Nakagawa and Schielzeth, 2013; Nakagawa et al., 2017).

## 3 Results

### 3.1 Representation of teacher-student relationship

#### 3.1.1 Teachers’ perspective

Table 2 presents the descriptive analyses performed on teachers’ responses to the STRS measure, in its two administered versions. In line with hypotheses A and B, we explored whether the diagnostic label and teacher-related variables shaped teachers’ perceptions of the relationship and their educational stance.

**Table 2:** Distributions of responses to STRS subscales and mean responses.

STRS subscales and pupils groups	n	Min	Max	M	SD	sk	ku	Mean response (Min = 1; Max = 5)
STRS conflict (10 Items)								
SLD pupils	65	10	40	16	6.62	1.28	1.41	1.6
WD pupils	65	10	37	16	6.99	1.37	1.13	1.6
STRS closeness (8 Items)								
SLD pupils	65	18	40	29.7	5	−0.11	−0.53	3.7
WD pupils	65	10	37	26.2	5.87	−0.64	−0.01	3.3
STRS dependency (4 Items)								
SLD pupils	66	4	15	7.6	2.86	0.82	−0.18	1.9
WD pupils	65	4	14	6.3	2.23	1.40	2.32	1.6

STRS Closeness (SLD pupils) was negatively correlated with weekly teaching hours (*r* = −0.25, *p* = 0.04), indicating that the greater the number of work hours, the less closeness was perceived in the relationship with an SLD pupil. The same dimension of Closeness with a WD pupil was positively correlated with years of teaching (*r* = 0.24, *p* = 0.05), showing a marginally significant association. With WD pupils, the STRS Dependency dimension was positively correlated with hours of teaching per week (*r* = 0.25, *p* = 0.04). These three associations were further examined through separate LMMs, each including teaching experience (years of teaching) and weekly hours spent with pupils as fixed effects and school as a random intercept. Neither the effect of teachers’ work experience on Closeness (WD pupils), nor that of weekly hours spent on Closeness (SLD pupils) yielded significant results. Thus, only the LMMs that showed significant effects are reported in detail. Specifically, for Closeness with WD pupils, the number of weekly hours spent with the pupil was a significant predictor (cf. M1, Table 3), indicating that more contact time was associated with higher perceived Closeness in the relationship with a WD pupil. Finally, Dependency scores related to pupils without a diagnosis were also significantly predicted by the weekly hours spent with the pupil (cf. M2, Table 3), suggesting that teachers perceive undiagnosed pupils as more dependent when they spend more time with them.

**Table 3:** LMMs examining STRS closeness and STRS dependency across WD and SLD pupils (school- and teacher-level random intercepts).

M	Predictor	Outcome	n	b	SE	t	df	p	Null model ICC		R^2^_m_	R^2^_c_	95% CI [low, high]
									School	Teacher			
1	Hours (WD)	STRS closeness (WD)	64	0.26	0.12	2.1	59.1	0.038	0.39	/	8.9%	38.8%	[0.02, 0.5]
2	Hours (WD)	STRS dependency (WD)	64	0.19	0.04	4.3	61	< 0.001	0*	/	22.7%	NA	[0.1, 0.28]
3	Interaction: hours (WD) x BP_F3	STRS dependency (WD)	63	−0.04	0.02	−2.2	59	0.032	0*	/	29.7%	NA	[−0.07, −0.004]
4	Weekly Working Hours	BP-F2	63	0.18	0.09	2	59.4	0.049	0.21	/	5.9%	23.8%	[0.004, 0.36]
5	SLD status (WD/SLD)	STRS closeness	130	3.64	0.81	4.5	62.9	< 0.001	0.08	0.07	10%	36.8%	[2.06, 5.21]
6	SLD status (WD/SLD)	STRS dependency	131	1.35	0.34	4	64.1	< 0.001	0*	0.35	11.2%	NA	[0.7, 2.01]

M: Model number. Each model (M1–M5) is numbered to facilitate cross-referencing between the text and this table; Hours (WD): weekly hours spent with the specific WD pupils; BP-F2: score on second factor of Bleak Pedagogy Scale, “Education of children over time”; BP-F3: score on third factor of Bleak Pedagogy Scale, “Methods of Bleak Pedagogy”; *: The null model returned ICC = 0, indicating no variance due to school-level clustering; /: Teacher could not be included as a random intercept due to insufficient variance; NA: Not Applicable; the analysis did not yield a conditional R^2^ because the random intercept explained no variance beyond the fixed effects.

Given these results, we explored through LMMs whether teachers’ endorsement of Adultcentrism and Bleak Pedagogy moderated the associations between time spent with the WD pupil and STRS-perceived Closeness or Dependency. This analysis further contributed to the investigation of hypothesis D. The only significant interaction emerged for BP’s methods subscale (cf. M3, Table 3), suggesting that the more teachers endorsed traditional methods of discipline and authority, the less they perceived increased contact time with undiagnosed pupils as indicating Dependency. The null model for this outcome, involving again the Dependency subscale of the STRS, also returned an *ICC* = 0, indicating no variance attributable to school-level clustering.

Regarding educational stance and its association with teacher-related variables, we found that BPO (*r* = −0.28, *p* = 0.034) and BP “Values of Bleak Pedagogy” factor (*r* = −0.26, *p* = 0.046) were negatively correlated with years of teaching. Conversely, BP “Education of children over time” factor, which reflects a nostalgic attitude towards old-fashioned practices considered effective and useful, was positively correlated with teachers’ working hours per week (*r* = 0.26, *p* = 0.042). To further test these associations, LMMs were fitted including a random intercept for school: results did not confirm the association between years of teaching and scores on BPO or BP’s values, but a positive association that approached significance was found between teaching hours per week and “Education of children over time” (cf. M4, Table 3).

Moreover, paired-sample *t*-tests highlighted two important differences. Firstly, teachers scored significantly higher (*M* = 29.7, *SD* = 5) on STRS Closeness when referring to an SLD pupil, than when considering a WD pupil (*M* = 26.2, *SD* = 5.9): *t*(63) = 4.708, *p* < 0.001 (95% CI = 2.18–5.39), *d* = 0.59. Secondly, teachers tended to perceive higher levels of dependency in the relationship when a pupil with SLD was considered (*M* = 7.6, *SD* = 2.86) compared to a pupil without a diagnosis but with poor school performance (*M* = 6.3, *SD* = 2.23): *t*(64) = 4.131, *p* < 0.001 (95% CI = 0.71–2.05), *d* = 0.51. To verify whether these dimensions of the pupil-teacher relationship varied depending on the student’s diagnostic status (hypothesis A), two LMMs were conducted: one for the Closeness subscale and one for Dependency. In both models, teachers and schools were treated as random factors to account for the nested structure of the data. Results showed that Closeness scores were significantly higher when teachers described their relationship with a pupil with an SLD compared to one without a diagnosis (cf. M5, Table 3). A similar pattern was found for the Dependency subscale, where higher scores emerged in the case of SLD pupils (cf. M6, Table 3).

#### 3.1.2 Pupils’ perspective

In accordance with our first objective (hypotheses A and B), we examined children’s perceptions of the teacher-pupil relationship and potential differences based on gender, age group, and diagnostic status. Table 4 presents the distributions of children’s responses on each of the three STRQ subscales, together with internal consistency coefficients (Cronbach’s *α*, *r.drop*), which were satisfactory across all three STRQ subscales.

**Table 4:** Distributions of responses to STRQ subscales and mean responses.

Scale	n	Min	Max	M	SD	sk	ku	Mean response (min = 1; max = 5)	Cronbach’s α		r.drop (mean)	
									Younger	Older	Younger	Older
STRQ closeness (7 items)	74	7	28	20.4	6.2	−0.45	−1.7	2.9	0.875	0.799	0.682	0.569
STRQ dissatisfaction (4 items)	73	4	16	7.8	3.6	0.92	−0.28	2	0.829	0.835	0.664	0.673
STRQ bond with school (6 items)	69	6	24	17.6	4.7	−0.63	−0.67	2.9	0.762	0.742	0.511	0.481

Descriptive statistics refer only to matched cases where both teacher and pupil data were available.

Distribution data by SLD/WD status and age group (younger/older) were not reported, as no differences were observed across these subgroups. Nonetheless, differences in scores were visible between males and females, considering the whole sample and the younger children subgroup (cf. Table 5).

**Table 5:** Male/female differences in scores on the three STRQ subscales.

Subscale	Paired differences								
	Highest M score	M_m_ - M_f_	SED	95% CI of the difference		t	df	*p*	Cohen’s d
				Lower	Upper				
All children									
STRQ closeness	Females	−4.6	0.72	−7.1	−2	−3.5	69	0.001	- 0.79
STRQ dissatisfaction	Males	2.9	2.9	1.4	4.3	3.98	71	0.000	0.85
STRQ bond with School	Females	−2.4	0.56	−4.7	−0.16	−2.15	47	0.037	−0.54
Older children subgroup									
STRQ closeness	Females	−2.3	1.04	−6.8	2.2	−1.1	16	0.296	−0.41
STRQ dissatisfaction	Males	2.2	0.7	−0.05	5	1.7	17	0.104	0.62
STRQ bond with School	Females	−0.1	0.8	−5.8	5.6	−0.04	7	0.970	−0.02
Younger children subgroup									
STRQ closeness	Females	−6	1	−9.4	−2.5	−3.5	41	0.001	−1.03
STRQ dissatisfaction	Males	3.2	0.5	1.4	5.1	3.6	37	0.001	1.03
STRQ bond with School	Females	−3.5	0.8	−6.2	−0.8	−2.6	38	0.013	−0.77

As shown, females reported significantly higher perceived closeness with teachers and positive attachment to the school, whereas males showed higher levels of Dissatisfaction. These results were also true for the younger children subgroup.

To further examine whether children’s responses to STRQ varied as a function of their gender, three LMMs were estimated including random intercepts for teacher and school. No significant main effect of gender was found on the three factors of STRQ, namely Closeness (cf. M1, Table 6), Dissatisfaction (cf. M2, Table 6), and Bond with School (cf. M3, Table 6). Although gender did not emerge as a significant predictor on any STRQ dimension, the analyses provided valuable insights into the variance structure of the data. Children’s scores on the Dissatisfaction subscale appear to be influenced by contextual factors: the high conditional R^2^ (96.7%) and moderate clustering at the school (ICC = 0.69) and teacher levels (ICC = 0.28) suggest that children’s dissatisfaction reflects the environment they experience rather than their gender. For completeness, given the pattern found for teachers regarding weekly hours, we also tested whether pupils’ STRQ scores were linked to the time the teacher reported spending with the specific child, but no significant correlations emerged, either for SLD or WD pupils. This may be due to the fact that children completed the STRQ with reference to their entire group of teachers, so this analysis might prove useful in future studies with individually matched teacher-pupil data.

**Table 6:** LMMs for children gender effects on STRQ subscales (school- and teacher-level random intercepts).

M	Predictor	Outcome	n	b	SE	t	df	*p*	Null model ICC		R^2^_m_	R^2^_c_	95% CI [low, high]
									School	Teacher			
1	Gender	STRQ closeness	16	3.38	3.89	0.9	14	0.4	0*	0*	4.8%	NA	[−4.24, 11.01]
2	Gender	STRQ dissatisfaction	15	−1.2	1.54	−0.8	7.2	0.46	0.69	0.28	2.5%	96.7%	[−4.22, 1.83]
3	Gender	STRQ bond with school	10	3.4	3.06	1.1	8	0.3	0*	0*	12.1%	NA	[−2.6, 9.4]

M: Model number. Each model (M1–M3) is numbered to facilitate cross-referencing between the text and this table; *: The null model returned ICC = 0, indicating no variance due to school-level or teacher-level clustering; NA: Not Applicable; the analysis did not yield a conditional R^2^ because the random intercept explained no variance beyond the fixed effects.

#### 3.1.3 Associations between teachers’ and pupils’ perspective on their relationship

In order to fully address hypotheses A and B, we investigated the associations between teachers’ and pupils’ perceptions of the relationship’s quality, although participants were not matched at the individual level, so the associations should be interpreted as group-level tendencies.

STRQ Dissatisfaction (SLD pupils) was positively correlated with STRS Conflict (SLD pupils) *r* = 0.4, *p* = 0.012, and with STRS Dependency (SLD pupils) *r* = 0.33, *p* = 0.025. To account for clustering by school, a LMM was fitted including a random intercept for school. *ICC* and *R*^2^ values are reported collectively, as the predictors were tested within a single model. A random intercept for teacher was not included, as each teacher contributed only one data point for the pupil with SLD, making it impossible to estimate teacher-level variance. Results showed that STRS Conflict significantly predicted higher levels of STRQ Dissatisfaction, while STRQ Dependency did not (cf. Table 7). These results should be interpreted with caution, as they reflect associations between aggregated teacher and student perceptions across schools, rather than matched dyadic data. Nonetheless, the conditional *R*^2^ of 63.1% and the *ICC* of 0.48 suggest that a considerable portion of variance in pupils’ dissatisfaction is linked to school-level factors, highlighting the role of context even when individual predictors are not significant.

**Table 7:** LMM examining STRS conflict and STRS dependency as predictors of STRQ dissatisfaction (school-level random intercept).

Predictor	Outcome	n	b	SE	t	df	p	Null model ICC (School)	R^2^_m_	R^2^_c_	95% CI [low, high]
STRS conflict (SLD)	STRQ dissatisfaction (SLD)	45	0.24	0.08	3	41.1	0.005	0.48	21.4%	63.1%	[0.08, 0.39]
STRS dependency (SLD)	STRQ dissatisfaction (SLD)	45	0.1	0.17	0.6	35.3	0.56				[−0.23, 0.43]

### 3.2 Teachers’ beliefs on SLD

Table 8 summarizes the descriptive analyses conducted on the results of the RADSA instrument, providing an initial overview of teachers’ attitudes and beliefs on SLD diagnosis. In pursuit of the second objective, aimed at deepening the understanding of teacher–pupil relationship quality by relating it – among other factors – to teachers’ beliefs about SLD diagnosis, we proceeded to explore the RADSA dimensions and their associations with teacher-related variables.

**Table 8:** RADSA: minimum and maximum possible scores, characteristics of the distribution of each subscale and mean responses.

Sections and factors	n	Min score	Max score	M	SD	sk	ku	Mean response (min = 1; max = 4)
Section 1: diagnoses increase								
Medicalization (7 items)	64	11	21	16.2	2.9	−0.142	−0.959	2.3
System-level causes (7 items)	65	15	27	21.9	2.7	−0.138	−0.351	3.1
How children are raised nowadays (4 items)	64	5	16	10.7	2	0.121	0.336	2.7
Section 2: peers								
Complaints about facilitations (6 items)	64	6	21	11.3	3.5	0.539	−0.179	1.8
Attention to emotions and needs (6 items)	65	14	23	18.2	2	−0.010	−0.768	3
Fairness of evaluation (5 items)	65	8	17	13.3	2	0.183	−0.386	2.7
Section 3: parents								
Roles and information (5 items)	64	13	20	16.6	1.8	0.348	−0.653	3.3
Diagnosis as alibi (4 items)	63	6	16	10.9	2.1	−0.008	−0.197	2.7
Parents’ negative reactions to SLD diagnosis (3 items)	63	4	12	8.2	1.6	0.312	0.339	2.7
Section 4: teachers								
Diagnosis usefulness (3 items)	65	3	11	7.2	2	−0.209	−0.171	2.4
Teacher positioning (6 items)	64	13	25	18.1	2.1	0.55	0.814	3
Strengths and weaknesses (7 items)	65	19	28	23.4	2.5	0.033	−1.18	3.3

Diagnoses increase: “Increase of SLD diagnoses” section; Peers: “Peer group” section; Parents: “Parents’ attitudes towards SLD diagnosis” section; Teachers: “The moment in which the teacher refers parents to an SLD specialist” section.

Some noteworthy correlations were found between this instrument and the sample’s characteristics. RADSA “Attention to the class emotions and individual needs” (*r* = 0.32, *p* = 0.009), “Roles and information” (*r* = 0.28, *p* = 0.023), “Fairness of evaluation” (*r* = 0.25, *p* = 0.042) were positively correlated with teachers’ age. Consistently, RADSA “Fairness of evaluation” was also positively related to years of teaching (*r* = 0.37, *p* = 0.003). Conversely, RADSA “Diagnosis as alibi” was negatively correlated (*r* = −0.26, *p* = 0.044) with years of teaching.

To test whether these correlations remained when accounting for school-level clustering, four LMMs were estimated including random intercepts for school. Results confirmed significant effects of teacher age on “Attention to the class emotions and individual needs” (cf. M1, Table 9) and “Roles and information” (cf. M2, Table 9). In addition, years of teaching predicted higher scores on “Fairness of evaluation” (cf. M3, Table 9) and lower scores on “Diagnosis as alibi” (cf. M4, Table 9). These findings also reveal that the explained variance is not negligible, particularly for “Roles and information” (*R*^2^_c_ = 37.9%), where school-level differences (*ICC* = 0.33) play a significant role. This suggests that teacher-related variables interact with school contexts in shaping these specific beliefs about SLD diagnosis.

**Table 9:** LMMs: significant effects of teacher age and experience on RADSA dimensions (school-level random intercept).

M	Predictor	RADSA’s subscale (outcome)	n	b	SE	t	df	p	Null model ICC (School)	R^2^_m_	R^2^_c_	95% CI [low, high]
1	Age-T	Attention to emotions and needs	62	0.11	0.05	2.4	61.6	0.018	0.09	11.1%	20.1%	[0.02, 0.2]
2	Age-T	Roles and information	59	0.12	0.04	2.8	58.2	0.006	0.33	10.9%	37.9%	[0.03, 0.2]
3	Exp	Fairness of evaluation	61	0.07	0.03	2.3	60.9	0.027	0.02	13.2%	16.6%	[0.01, 0.13]
4	Exp	Diagnosis as alibi	60	−0.07	0.03	−2.1	59.9	0.038	0.14	7.1%	15.1%	[−0.14, −0.01]

M: Model number. Each model (M1–M4) is numbered to facilitate cross-referencing between the text and this table; Age-T: Teachers’ age. Exp: years of teaching (experience).

#### 3.2.1 Associations of teachers’ beliefs on SLD with the educational stance and practices

Table 10 illustrates descriptive statistics of the Adultcentrism paradigm and the Bleak Pedagogy educational stance in the present sample. To address hypothesis C, we investigated how adult-centered tendencies and authoritarian educational values and practices align with specific RADSA subscales.

**Table 10:** Adultcentrism scale for teachers, Bleak Pedagogy scales and subscales, minimum and maximum scores, mean total scores, distribution of responses and mean responses.

Section or subscale	n	Min score	Max score	M	SD	sk	ku	Mean response (min = 1; max = 4)
Bleak Pedagogy Observation (24 items)	59	37	73	55.6	8.4	−0.24	−0.811	2.3
Values of Bleak Pedagogy (11 items)	60	18	42	29.6	5	0.076	0.139	2.7
Education of children over time (5 items)	63	8	19	13.6	2.6	0.15	−0.294	2.7
Methods of Bleak Pedagogy (7 items)	63	7	16	10.2	2.4	0.695	−0.479	1.5
Adultcentrism Scale for Teachers (9 items)	64	13	25	18.8	2.34	0.014	0.097	2.1

The correlations observed between the RADSA instrument, the ADT scale, and BPO scale, were informative (cf. Table 11). Both Adultcentrism and Bleak Pedagogy positively correlated with two subscales of RADSA section concerning the increase of SLD diagnoses (“System-level causes” and “How children are raised nowadays”).

**Table 11:** Summary of Pearson product–moment correlations, between RADSA subscales, Bleak Pedagogy subscales and Adultcentrism scale for teachers.

Scale	ADT	BPO	BP-F1	BP-F2	BP-F3
Section 1: diagnoses increase					
Medicalization	−0.05	0.1	−0.01	0.18	0.16
System-level causes	0.33***	0.30**	0.36***	0.26*	−0.05
How children are raised nowadays	0.37***	0.29*	0.38***	0.13	−0.03
Section 2: peers					
Complaints about facilitations	0.16	0.40***	0.40***	0.36***	0.07
Attention to emotions and needs	0.35***	−0.05	0.04	0.06	−0.31**
Fairness of evaluation	0.01	−0.35***	−0.30**	−0.31**	−0.23
Section 3: parents					
Roles and information	0.32**	0.03	0.19	−0.01	−0.26*
Diagnosis as alibi	0.33***	0.35***	0.35***	0.32**	0.04
Parents’ negative reactions to SLD diagnosis	0.20	0.36***	0.34***	0.26*	0.14
Section 4: teachers					
Diagnosis usefulness	0.08	0.24	0.21	0.33***	−0.01
Teacher positioning	0.24	0.38***	0.36***	0.25	0.19
Strengths and weaknesses	0.07	−0.44***	−0.32**	−0.25*	−0.44***

*p < 0.05, **p < 0.03, ***p < 0.01. Diagnoses increase: “Increase of SLD diagnoses” RADSA section; Peers: “Peer group” RADSA section; Parents: “Parents’ attitudes towards SLD diagnosis” RADSA section; Teachers: “The moment in which the teacher refers parents to an SLD specialist” RADSA section. ADT: score on “Adultcentrism Scale for Teachers”; BPO: total score on “Bleak Pedagogy Observation” section; BP-F1: score on first factor of Bleak Pedagogy Scale, “Values of Bleak Pedagogy”; BP-F2: score on second factor of Bleak Pedagogy Scale, “Education of children over time”; BP-F3: score on third factor of Bleak Pedagogy Scale, “Methods of Bleak Pedagogy”.

As far as the “Peer group” RADSA section is concerned, higher scores on BPO, and two of its subscales – “Values of Bleak Pedagogy” and “Education of Children over Time” – were associated with teacher’s impression that more complaints are made in class about the accommodations available to a pupil with SLD by their peers, and to their conviction that it would be unjust to come up with different tests and homework for SLD pupils by adapting them to each individual’s specific abilities. Adultcentrism positively correlated with the idea that the teacher has to actively promote an inclusive classroom climate, by paying attention to emotions and individual needs. On the contrary, this RADSA dimension was negatively correlated with BP’s methods.

With regard to the third RADSA section “Parents,” it was found that the higher the accordance with Bleak Pedagogy and Adultcentrism, the more parents were seen as adults who use their child’s diagnosis as an alibi for every weakness or problem. BPO also positively correlates with the idea that parents have excessively negative reactions to SLD diagnoses. As the scores on Bleak Pedagogy’s methods increase, agreement with the idea that parents need more training about SLDs decreases. On the other hand, teachers scoring higher on Adultcentrism were more likely to believe that parents need more specific training about SLDs and about the role of each professional involved in the diagnostic process.

Lastly, the more teachers perceived their role as penalized compared to the other professionals involved in the assistance and diagnosis of an SLD case, the more agreement with the Bleak Pedagogy construct was detected, especially with BP’s values. A higher agreement with Bleak Pedagogy and all its subscales was instead negatively related to the tendency to focus on the teacher’s and the child’s weaknesses, problems, and lack of resources, when dealing with SLDs (RADSA “Strengths and weaknesses”). Moreover, BP subscale “Education of children over time” correlated in a positive direction with the opinion that the diagnosis is not particularly useful for teachers when dealing with a pupil with an SLD (RADSA “Diagnosis usefulness”).

In order to test more rigorously the associations between RADSA dimensions and the constructs of Adultcentrism and Bleak Pedagogy, a series of LMMs were run (cf. Table 12): results of these analyses confirmed the pattern observed in the bivariate correlations. The I*CC* and *R*^2^ values further indicate that, while the associations between Adultcentrism, Bleak Pedagogy, and certain RADSA dimensions are mainly driven by individual teacher beliefs (low *ICC* and *R*^2^_c_), some dimensions appear to be more sensitive to school-level factors, as suggested by higher conditional *R*^2^ values.

**Table 12:** LMMs with Adultcentrism or Bleak Pedagogy as predictors and RADSA subscales as outcomes (school-level random intercept).

Predictor	RADSA’s subscale (outcome)	n	b	SE	t	df	p	Null model ICC (School)	R^2^_m_	R^2^_c_	95% CI [low, high]
Section 1: diagnoses increase											
ADT	System-level causes	64	0.35	0.13	2.7	57	0.009	0.24	9.5%	28.8%	[0.1, 0.6]
BP-F1	System-level causes	60	0.17	0.06	2.7	56	0.01	0.24	9.9%	42.1%	[0.04, 0.29]
ADT	How children are raised nowadays	63	0.32	0.10	3.1	61	0.003	0*	13.6%	NA	[0.12, 0.53]
BPO	How children are raised nowadays	58	0.07	0.03	2.2	56	0.03	0*	8%	NA	[0.01, 0.13]
BP-F1	How children are raised nowadays	59	0.15	0.05	3.1	57	0.003	0*	14.1%	NA	[0.06, 0.25]
Section 2: peers											
BPO	Complaints about facilitations	58	0.17	0.05	3.3	56	0.002	0*	15.8%	NA	[0.07, 0.27]
BP-F1	Complaints about facilitations	59	0.28	0.09	3.3	57	0.002	0*	15.7%	NA	[0.11, 0.45]
BP-F2	Complaints about facilitations	62	0.47	0.16	3	60	0.004	0*	12.8%	NA	[0.16, 0.78]
ADT	Attention to emotions and needs	64	0.29	0.1	2.9	61.3	0.005	0.1	11.6%	15.4%	[0.09, 0.49]
BP-F3	Attention to emotions and needs	64	−0.26	0.09	−2.7	50.5	0.009	0.1	9.6%	24.7%	[−0.44, −0.07]
BPO	Fairness of evaluation	59	−0.08	0.03	−2.8	48.4	0.007	0.02	12.3%	13.1%	[−0.14, −0.03]
BP-F1	Fairness of evaluation	60	−0.12	0.05	−2.3	51.8	0.026	0.02	8.3%	11.7%	[−0.21, −0.02]
BP-F2	Fairness of evaluation	63	−0.23	0.09	−2.5	61	0.014	0.02	9.3%	NA	[−0.40, −0.05]
Section 3: parents											
ADT	Roles and information	63	0.20	0.09	2.3	52.5	0.025	0.33	6.8%	32.1%	[0.03, 0.38]
BP-F3	Roles and information	63	−0.18	0.08	−2.1	47.7	0.040	0.33	5.3%	33.6%	[−0.34, −0.01]
ADT	Diagnosis as alibi	62	0.31	0.11	2.9	57.6	0.005	0.14	11.7%	24.5%	[0.10, 0.52]
BPO	Diagnosis as alibi	57	0.08	0.03	2.7	52.2	0.010	0.14	11.7%	19%	[0.02, 0.15]
BP-F1	Diagnosis as alibi	58	0.14	0.05	2.8	55.1	0.008	0.14	12.1%	22.8%	[0.04, 0.25]
BP-F2	Diagnosis as alibi	61	0.23	0.1	2.4	56.4	0.018	0.14	9.1%	12.8%	[0.05, 0.42]
BPO	Parents’ negative reactions to SLD diagnosis	57	0.07	0.02	2.8	55	0.007	0*	12.4%	NA	[0.02, 0.12]
BP-F1	Parents’ negative reactions to SLD diagnosis	58	0.11	0.04	2.7	56	0.009	0*	11.3%	NA	[0.03, 0.19]
BP-F2	Parents’ negative reactions to SLD diagnosis	61	0.16	0.08	2.1	59	0.041	0*	6.8%	NA	[0.01, 0.31]
Section 4: teachers											
BPO	Diagnosis usefulness	59	0.06	0.03	2.1	56.8	0.041	0.21	7.1%	25.6%	[0.004, 0.12]
BP-F2	Diagnosis usefulness	63	0.25	0.09	2.8	61	0.007	0.21	11.2%	26.9%	[0.08, 0.43]
BPO	Teacher positioning	58	0.09	0.03	3	51.2	0.004	0.13	13.9%	17.8%	[0.03, 0.15]
BP-F1	Teacher positioning	59	0.15	0.05	2.9	52.5	0.006	0.13	12.6%	17.6%	[0.05, 0.25]
BPO	Strengths and weaknesses	59	−0.14	0.04	−3.8	40.4	0.001	0.05	20.1%	23.8%	[−0.21, −0.07]
BP-F1	Strengths and weaknesses	60	−0.16	0.06	−2.5	58	0.014	0.05	9.8%	NA	[−0.28, −0.04]
BP-F2	Strengths and weaknesses	63	−0.24	0.18	−2	53.2	0.047	0.05	6.3%	10.5%	[−0.47, −0.01]
BP-F3	Strengths and weaknesses	64	−0.46	0.12	−3.9	62	0.000	0.05	19.2%	NA	[−0.69, −0.22]

ADT: score on “Adultcentrism Scale for Teachers”; BPO: total score on “Bleak Pedagogy Observation” section; BP-F1: score on first factor of Bleak Pedagogy Scale, “Values of Bleak Pedagogy”; BP-F2: score on second factor of Bleak Pedagogy Scale, “Education of children over time”; BP-F3: score on third factor of Bleak Pedagogy Scale, “Methods of Bleak Pedagogy”; Diagnoses increase: “Increase of SLD diagnoses” RADSA section; Peers: “Peer group” RADSA section; Parents: “Parents’ attitudes towards SLD diagnosis” RADSA section; Teachers: “The moment in which the teacher refers parents to an SLD specialist” RADSA section. *: The null model returned ICC = 0, indicating no variance due to school-level clustering; NA: Not Applicable; the analysis did not yield a conditional R^2^ because the random intercept explained no variance beyond the fixed effects.

### 3.3 Teachers’ beliefs on SLD and the complexity of the teacher-student relationship

To further pursue the second objective (hypotheses C and D), we examined the interplay between teachers’ beliefs about SLD, their educational stance, and the perceived quality of pupil-teacher relationships, as reported by both teachers and pupils. It should be emphasized once again that these associations reflect group-level trends, as teachers and students were not matched at the individual level. Noteworthy results emerged from the exploration of correlations between children’s responses on STRQ and teachers’ beliefs about SLD diagnoses. First, only the correlations involving responses of SLD pupils reached statistical significance, except for one case; the others were not significant. STRQ Dissatisfaction (SLD pupils) rose alongside (*r* = 0.34, *p* = 0.024) RADSA “System-level causes,” the teachers’ belief that the increase of SLD cases in schools is ascribable to society, parents’ chaotic life, law regulations on SLD, etc. Secondly, STRQ Dissatisfaction (SLD pupils) was also positively related to teachers’ agreement with RADSA “Attention to classroom emotions and to individual needs” dimension (*r* = 0.35, *p* = 0.017). Pupils with SLD revealed more Dissatisfaction (STRQ) when their teacher showed more agreement with the idea that parents need more specific training on SLDs as well (RADSA “Roles and information”): (*r* = 0.31, *p* = 0.038). In addition, the more teachers agreed with the belief that the SLD diagnosis is not particularly useful in itself to deal with a pupil with an SLD (RADSA “Diagnosis usefulness”), the less the latter expressed Dissatisfaction in STRQ (*r* = −0.33, *p* = 0.025). Regarding Bleak Pedagogy, higher scores on “Values of Bleak Pedagogy” were associated with pupil (SLD) expressing lower levels of closeness in the relationship (*r* = −0.41, *p* = 0.008) and of positive bonds with the school context (*r* = −0.34, *p* = 0.032), which also negatively correlated with Adultcentrism (*r* = −0.32, *p* = 0.038). Moreover, teachers’ perception of closeness in the relationship (STRS) considering a pupil with SLD was negatively associated with BP’s methods (*r* = −0.37, *p* = 0.003). Finally, Adultcentrism and perceived Dependency (STRS) were positively related (*r* = 0.31, *p* = 0.013) when considering SLD pupils, and this was the only trend also confirmed across the whole sample of children (*r* = 0.21, *p* = 0.016).

To further examine the associations described above, a series of LMMs were fitted including a random intercept only for school, since each teacher provided only one evaluation per pupil. Below, only the models that reached statistical significance are reported.

Results showed that teachers’ agreement with “Diagnosis usefulness” dimension significantly predicted STRQ Dissatisfaction scores in SLD pupils (cf. M1, Table 13). Children’s Dissatisfaction was also positively predicted by teachers’ emphasis on the “Roles and Information” dimension (cf. M2, Table 13). Regarding Bleak Pedagogy, higher teacher endorsement of “Values of Bleak Pedagogy” predicted lower STRQ Closeness (cf. M3, Table 13) and weaker Bond with School in SLD pupils (cf. M4, Table 13). Similarly, higher Adultcentrism association with lower STRQ Bond with School scores was confirmed (cf. M5, Table 13).

**Table 13:** Significant LMMs examining the associations between teachers’ beliefs on SLD, educational stance, and relationship quality (school-level random intercept).

M	Predictor	Outcome	n	b	SE	t	df	p	Null model ICC (School)	R^2^_m_	R^2^_c_	95% CI [low, high]
1	Diagnosis usefulness	STRQ dissatisfaction (SLD)	45	−0.76	0.27	−2.8	37.1	0.007	0.47	11.7%	65.8%	[−1.2, −0.23]
2	Roles and information	STRQ dissatisfaction (SLD)	45	0.64	0.29	2.2	38.5	0.036	0.47	7.7%	51.9%	[0.06, 1.21]
3	BP-F1	STRQ closeness (SLD)	41	−0.48	0.18	−2.7	38.7	0.012	0.17	14.5%	37.4%	[−0.8, −0.14]
4	BP-F1	STRQ bond with school (SLD)	39	−0.27	0.11	−2.4	36	0.02	0.19	13.1%	39%	[−0.4, −0.05]
5	ADT	STRQ bond with school (SLD)	42	−0.58	0.27	−2.1	40	0.038	0.19	10.1%	NA	[−1.1, −0.03]
6	BP-F3	STRS closeness (SLD)	63	−0.73	0.24	−3.1	61	0.003	0.06	13.6%	NA	[−1.1, −0.27]
7	BP-F1	STRS closeness (SLD)	58	0.79	0.24	3.3	50	0.002	0.06	1.7%	19%	[0.32, 1.26]
8	BP-F3	BP-F2	63	0.38	0.12	3.1	49.5	0.003	0.21	12.3%	28.6%	[0.14, 0.62]
9	ADT	STRS dependency	64	0.48	0.22	2.2	61.5	0.033	0*	7%	10.1%	[0.05, 0.91]

M: Model number. Each model (M1–M9) is numbered to facilitate cross-referencing between the text and this table. * The null model returned ICC = 0, indicating no variance due to school-level clustering. NA: Not Applicable; the analysis did not yield a conditional R^2^ because the random intercept explained no variance beyond the fixed effects; ADT: score on “Adultcentrism Scale for Teachers. BPO: total score on “Bleak Pedagogy Observation” section; BP-F1: score on first factor of Bleak Pedagogy Scale, “Values of Bleak Pedagogy”; BP-F2: score on second factor of Bleak Pedagogy Scale, “Education of children over time”; BP-F3: score on third factor of Bleak Pedagogy Scale, “Methods of Bleak Pedagogy”.

Teachers’ perception of the relationship quality with SLD pupils was also affected by their educational stance and practice: higher scores on “Methods of Bleak Pedagogy” were associated with lower perceived STRS Closeness (cf. M6, Table 13), but agreement with “Values of Bleak Pedagogy” predicted higher scores on Closeness perceived in the relationship (cf. M7, Table 13). Interestingly, while BP’s values were associated with higher perceived closeness, this pattern diverged from what was found for BP’s methods. This prompted us to focus on BP’s second factor (“Education of children over time”), which also reflects aspects of educational practice—although framed in temporal terms. Among all variables, the only one directly linked to both this second factor and pupils themselves was the weekly hours spent with them. This observation guided our further analysis: we tested whether agreement with BP’s methods would predict higher scores on “Education of children over time” (a nostalgic attitude towards old-fashioned methods), assuming that the way teachers manage time with pupils would reflect a consistent pattern shaped by those methods. The LMM confirmed this hypothesis of association: a higher agreement with BP’s methods predicted a more nostalgic attitude towards those same methods (cf. M8, Table 13). However, the mediation analysis testing the role of weekly hours as a mediator between BP’s methods and “Education of children over time” did not yield significant results (*ACME* = −0.005, *p* = 0.86), while the direct effect of BP’s methods remained significant (*ADE* = 0.421, *p* = 0.001).

Finally, higher Adultcentrism was confirmed as a predictor of higher STRS Dependency considering both SLD and WD pupils (cf. M9, Table 13). These results should be interpreted within the framework of partially overlapping samples, as they illustrate meaningful associations between beliefs and perceptions at the group level, without assuming matched identities between individual teachers and students. Taken together, the relatively high school-level *ICC*s and *R*^2^_c_ values observed for STRQ models (up to 65.8%)—compared to the minimal clustering in STRS—may suggest that pupils’ perceptions of relational quality are more context-dependent, whereas teachers’ self-perceptions appear comparatively stable. The recurring associations with Bleak Pedagogy-related predictors (M3, M4, M6; Table 13) and the potential influence of Adultcentrism (M5, M9; Table 13) could indicate that authoritarian educational stances and adult-centered attitudes play a role in shaping both relationship quality and pupils’ bonds with the school context.

## 4 Discussion

This study investigated teachers’ perceptions of the quality of their relationships with pupils diagnosed with SLD compared to low achievers without diagnosis, by considering a set of teacher-related factors (teaching experience and weekly working hours) and pupil-related characteristics (age and gender). Furthermore, we examined how these elements interact with broader constructs, such as labeling effects and educational stance, to provide a deeper understanding of the dynamics underlying teacher-student relationships. The quality of such relationship appears to be partly influenced by the presence of an SLD diagnosis, since both the perceived levels of Closeness and Dependency were significantly higher when teachers described the relationship with a pupil with an SLD, rather than with a pupil without a diagnosis but identified by the teacher as having poor school performance was taken into consideration. Previous studies have mainly compared pupils with and without an SLD diagnosis, focusing on differences in teacher-student relationships (e.g., Al-Yagon and Mikulincer, 2004; Murray and Greenberg, 2000; Zee et al., 2020) and exploring teachers’ implicit and explicit attitudes (Hornstra et al., 2010). To our knowledge, very few studies (e.g., Pasta et al., 2013) have directly compared pupils with a formal SLD diagnosis to low achievers without diagnosis. By adopting this finer distinction, our study contributes a novel perspective that helps disentangle the effects of diagnostic labeling from those related solely to academic performance. Our results align with both the primary expectations of the present research and the findings of previous studies. In fact, the STRS Closeness dimension is considered the most representative of a positive relationship (Sabol and Pianta, 2012), and it has been found that the presence and type of diagnosis can be a factor that may influence it (Pasta et al., 2013; Zee et al., 2020). However, this comparison should be interpreted with caution, as the group of WD pupils may include heterogeneous profiles, and the definition of low achievement was based on teachers’ subjective assessments rather than standardized criteria. Therefore, it is important to note that the comparison group may have included pupils with diverse and possibly undiagnosed difficulties, which could intervene in challenging the quality of the pupil-teacher relationship, thus diminishing the perception of closeness in a similar way to what was found for pupils with difficulties that had a greater impact at the behavioral level (Zee et al., 2020).

Although a bivariate correlation initially suggested a negative association between weekly teaching hours and perceived Closeness with SLD pupils, this effect was not confirmed by the LMM.

Instead, the significant effect of weekly hours spent with the WD pupil was confirmed as a predictor of higher levels of Dependency (STRS) and Closeness (STRS) perceived by the teacher. This pattern suggests that increased contact time may be associated with both enhanced relational involvement and a stronger perception of the pupil’s reliance on the teacher. However, the moderation effect of Bleak Pedagogy’s methods indicates that this interpretation is not uniform: the more teachers endorsed authoritarian educational styles, the less they interpreted increased time as a sign of Dependency. This moderation effect may reflect not a lack of relational engagement, but rather a specific normative view of educational responsibility, where closeness is valued and cultivated, yet interpreted through a lens of directive guidance and control – a stance that, if accepted by the other member of the relationship (i.e., the child), may indeed foster dependency without necessarily generating Dissatisfaction, as also suggested by the absence of significant associations with this STRQ subscale. This interpretation is consistent with Maturana and Dávila’s (2006) claim that adult control in the educational relationship generates dependence in children. And, as suggested by Florio et al. (2023), teachers who endorse Bleak Pedagogy’s methods may be genuinely convinced that increased involvement and directivity serve the child’s best interests. In this view, spending more time with a WD pupil does not necessarily imply fostering autonomy or shared agency, but rather ensuring protection, order, and “proper” development. In sum, such time investment may enhance relational closeness while simultaneously fostering a perception of the pupil as dependent – unless such dependency is reframed, by some degree of adherence to authoritarian educational stance, as appropriate receptivity to adult guidance. These interpretations remain provisional, as the mechanisms linking contact time with the pupil, perceived dependency, and educational stance may involve bidirectional dynamics or contextual factors beyond the scope of this study. Longitudinal research would be valuable to examine how these relationships evolve over time and to disentangle the direction of these effects.

Regarding years of experience, previous research has shown that teaching experience can influence teacher-student relationship perceptions (Zee and Koomen, 2017). In our study, however, no significant association was found between teaching experience and the quality of teacher-student relationships, suggesting that this link may depend on contextual factors or other variables not captured in the present design.

The finding that teachers report greater Closeness and Dependency in their relationships with SLD pupils compared to peers without a diagnosis, albeit with equally poor school performance, suggests that the diagnostic label might activate a more empathic, protective, or affiliative stance. The result concerning Closeness appears consistent with the literature emphasizing the positive impact of diagnosis as a relational “framework” (Zee et al., 2020; Pasta et al., 2013), helping teachers to interpret students’ difficulties through a lens that reduces blame and facilitates supportive behaviors. Regarding Dependency, the higher scores in relation to SLD pupils might reflect teachers’ increased willingness to provide help and guidance to students officially recognized as struggling. However, this interpretation warrants caution: over-involvement and perceptions of excessive need may also emerge, especially in classroom environments where the diagnosis becomes a cue for permanent vulnerability rather than a signal of support potential (cf. Pasta et al., 2013). In fact, prior work suggests that a formal diagnosis may alter teachers’ attributions, leading them to view academic difficulties as less changeable and more reflective of the pupil’s inherent limitations which can ultimately reinforce low expectations (Frohlich et al., 2020; Woodcock and Vialle, 2011). It has to be underlined that the cross-sectional nature of our data does not allow for determining such temporal direction of the associations we found.

Some noteworthy findings emerged from the exploration of associations between pupils’ and teachers’ representations of their relationship. Before delving into these results, it is worth recalling that internal consistency analyses supported the reliability of STRQ subscales across both younger and older children. All three subscales showed acceptable to good internal consistency, as indicated by Cronbach’s *α* and by mean corrected item-total correlation (*r.drop*), in line with recommended thresholds (Nunnally and Bernstein, 1994; Hair et al., 2019). These findings strengthen the reliability of the instrument even among younger pupils, despite the original validation of STRQ being conducted primarily on 4th and 5th grade students (Tonci et al., 2012).

Correlational analyses between children’s and teachers’ responses revealed that when teachers express that they perceive more Conflict and Dependency in their relationship with SLD pupils, the same pupils report more Dissatisfaction in their relationship with the teachers. This relationship was also confirmed by LMM analysis, showing that especially teacher-perceived conflict seems to play a predictive role toward perceived Dissatisfaction among SLD pupils. These findings align with the extensive literature emphasizing the protective role of positive teacher-student relationships (García-Rodríguez et al., 2023; Haldimann et al., 2023; Jederlund and von Rosen, 2023; Thornberg et al., 2022), particularly for pupils with diagnosed vulnerabilities (Re et al., 2021; Zee et al., 2020). They also support a systemic perspective, which conceptualizes both student and teacher as active constructors of the relationship quality (Pianta, 2001a).

Differently from a previous study that explored teacher-pupil relationship quality using a multi-informant approach (Zee et al., 2020), no significant differences emerged in our sample between SLD and WD pupils in children’s STRQ scores. This result should be considered in light of the mismatch between STRS and STRQ measures, which may partly explain the lack of differences observed as children reported on teachers in general while teachers referred to a specific pupil. It should be noted, however, that Zee and colleagues employed a different instrument – the “Student Perception of Affective Relationship with Teacher Scale” (SPARTS; Koomen and Jellesma, 2015)—and therefore the two studies are not fully comparable. Furthermore, it is important to consider that in our study the grouping into SLD and WD pupils was based on teachers’ subjective assessment, and that the sample did not include children who were perceived by their teachers as having no learning-related difficulties.

On the other hand, in line with the findings of previous studies (Koomen and Jellesma, 2015; La Grutta et al., 2023; Longobardi et al., 2016; Zee and Koomen, 2017), gender differences were found in the perception of the teacher-pupil relationship: female pupils reported significantly higher levels of perceived closeness in the relationship and positive attachment to the school, whereas male pupils expressed greater Dissatisfaction. This pattern remained evident among younger children subgroup. These results are consistent with previous findings suggesting that girls tend to describe more positive relationships with teachers, and a stronger affective bond with the school environment (Murray and Zvoch, 2011; Hamre and Pianta, 2001). In contrast, boys may be more likely to experience conflict and emotional distance in the relational climate, since they tend to express more externalizing emotions (e.g., anger) during middle childhood (Chaplin and Aldao, 2013). Moreover teachers—especially female teachers, though not exclusively – have been found to tend to report lower levels of closeness and/or higher levels of conflict in their relationships with male pupils (Quaglia et al., 2013; Spilt et al., 2012). Such dynamics could make the relational exchange challenging for both parts: if the teacher perceives the relationship as tense, this may be expressed through reduced warmth or patience, and the pupil, in turn, may feel less understood or supported, reinforcing dissatisfaction and disengagement from school environment. This interpretation remains hypothetical and should be tested in future research specifically designed to explore these reciprocal dynamics, particularly since the significance of these differences in paired comparisons was not confirmed by the LMMs: a main effect of gender was absent when accounting for the nested structure of the data. This may suggest that the observed gender-based differences could vary depending on teacher or school specific factors, and that inter-individual variability at higher levels may attenuate these group-level effects when modeled appropriately. Additional classroom or school-level variables, which were not measured here, might further explain these gender-related differences.

According to the second aim of this study, the resulting pattern of correlations, confirmed by LMMs, delineates the profile of a teacher who might struggle when navigating the complex relational and interpretative framework captured by RADSA, especially in presence of strong agreement with Adultcentrism and/or Bleak Pedagogy constructs. A consistent finding is that higher scores on these scales are associated with the belief that the rise in SLD diagnoses is driven by system-level issues, or changes in child-rearing practices. Such associations may reflect a skeptical attitude towards the legitimacy or utility of the diagnostic process itself, since it seems to be perceived as the by-product of social or cultural transformations rather than of individual needs. A possible explanation of this significant association is that this belief system may resonate with what Kimura et al. (2024) define as “authoritarian nostalgia,” that is, “a collective political memory that is painful because the past is perceived in positive and even idyllic ways.” (Kimura et al., 2024, p. 389). In educational settings, this outlook may manifest in a preference for disciplinary structures perceived as lost.

Further differences emerged between the Adultcentric and Bleak Pedagogy perspectives with respect to the perceived role of parents. While Adultcentrism was positively associated with the belief that parents need more training about SLDs and the diagnostic process, BP’s methods subscale showed a negative relationship with this same belief. These diverging directions may reflect the fact that Adultcentrism, as a more theoretical and responsibility-focused stance, promotes the idea that adults should be better informed and proactive. In contrast, agreement with traditional authoritarian methods may discourage collaborative views and portray parental involvement and information as either excessive or unnecessary. This is in line with the skeptical attitude towards the legitimacy or utility of the diagnostic process described above: if the rise in diagnoses is perceived as the result of social or cultural changes, rather than of an improved understanding of learning disorders, then the value of parent training itself may be called into question altogether. On the contrary, the total score on BPO (especially in its values and convictions regarding educational change over time, i.e., first and second factors) and the score on ADT were both positively related to the belief that parents use their child’s diagnosis as an alibi for any weakness or problem. Only BPO, however, was associated with the belief that parents tend to react negatively to SLD diagnoses. When considered together with the previous results, these views appear to reflect a rather critical conception of parenthood: parents are implicitly blamed for their children’s difficulties, either for working too much or for transmitting inadequate values through education, and are seen as reacting defensively to the diagnosis – using it not as a tool for understanding and supporting, but as a form of social protection that shifts the focus onto individual pathology. In this framework, when there is agreement with Adultcentrism and/or Bleak Pedagogy, the reasoning around SLDs seems to revolve more around parents than around the children themselves. This consideration may align with the blaming process that has already been noted in other studies where children’s difficulties (e.g., behavioral, social, emotional) are ascribed to inadequate parenting—particularly to mothers—characterized by chaotic or inconsistent practices (Broomhead, 2013; Francis, 2012; Harborne et al., 2004; Peters, 2012; Rogers, 2007) which ultimately conveys the idea that children are seen as not having been properly raised or taught how to behave, indeed frequent reference are made to ineffective parenting and lack of discipline (Broomhead, 2013).

These beliefs also extended to the perceived functioning of the school context. Teachers with higher scores on Bleak Pedagogy, particularly in its values and conception of changes in education over time, expressed stronger agreement with the idea that it is unfair to tailor evaluation methods to pupils with SLDs, and more often perceived complaints from classmates about the compensatory tools assigned to these pupils. This could signal a perception of inclusive practices as lacking legitimacy or producing friction within the classroom. Conversely, higher Adultcentrism was related to greater teachers’ attention to classroom emotions and individual needs. This result may be interpreted by considering the profile of an adult who is sensitive to relational dynamics, yet sees their own role as central in shaping an inclusive classroom climate: something that, from this perspective, would not spontaneously emerge among pupils without adult intervention. As a support for this interpretation, it is recalled that STRQ Dissatisfaction (SLD pupils) was also positively related to teachers’ agreement with the “Attention to classroom emotions and to individual needs” RADSA dimension. This somewhat paradoxical finding might reflect a mismatch between teachers’ declared sensitivity and pupils’ lived experiences, which may be perceived as emotional overregulation rather than authentic emotional recognition and support.

Finally, teachers who felt that their professional role is penalized compared to that of other specialists involved in the diagnostic process showed greater agreement with Bleak Pedagogy, especially in its values. This finding was also confirmed by LMMs and accompanied by lower endorsement of the need to focus on pupils’ or teachers’ strengths and resources when dealing with SLDs. Moreover, agreement with BPO and its first two factors (BP’s values and education of children over time) was associated with the belief that the diagnosis is not particularly useful for addressing SLDs.

Together, these patterns may suggest a broader sense of disillusionment and marginalization among teachers whose professional stance partially aligns with Bleak Pedagogy. Rather than identifying these positions as direct causes of dysfunction, it may be more appropriate to interpret them as signs of cultural misalignment: when teachers endorse adult-centered or nostalgic views shaped by past authoritarian educational norms, they may struggle to reconcile their professional identity with the collaborative and evolving logic required by inclusive education. In this light, resistance to contemporary practices does not stem solely from ideological rigidity, but may reflect deeper tensions related to teachers’ perceived lack of recognition, autonomy, or access to educational resources, especially when their role is felt to be marginal within multidisciplinary dynamics surrounding diagnosis and inclusion. Nevertheless, these interpretations remain tentative, as our design does not allow us to clarify the complex interplay between teachers’ beliefs, professional identity, and broader systemic dynamics. Other cultural or institutional factors, not directly assessed in this study, may also contribute to shaping these critical views toward diagnostic practices and inclusive policies.

Finally, several noteworthy results emerged from the associations between teachers’ beliefs and the quality of their relationship with pupils diagnosed with SLDs. Stronger endorsement of Bleak Pedagogy methods was associated with lower Closeness (STRS) perceived in the relationship by teachers, as well as with lower Closeness (STRQ) and weaker Bond with School as reported by the SLD pupils themselves. Higher scores on the values dimension of Bleak Pedagogy were also negatively related to SLD pupils’ sense of closeness with their teacher and their attachment to school. In addition, pupils’ Dissatisfaction increased when teachers scored higher on beliefs attributing the rise in diagnoses to system-level causes or advocating for more parental training. Conversely, pupils’ Dissatisfaction decreased when their teacher showed stronger agreement with the idea that the diagnosis is not particularly useful in itself, suggesting that certain critical attitudes toward the diagnosis may coexist with more favorable relational dynamics – possibly because they resonate with children’s own ideas about the meaning of diagnosis, an aspect not explored in the present study but worthy of further investigation. Aside from this particular finding, the overall direction of the other patterns concerning teachers’ educational stance and their perception of relationship quality may suggest that some attitudes, although aimed at promoting adult responsibility or systemic awareness, may be perceived by pupils as distancing or misaligned with their needs.

From a different angle, teachers scoring higher on BP’s values reported a stronger perception of closeness in the relationship with SLD pupils, pointing to a possible disconnection between teachers’ and pupils’ experiences. One possible interpretation is that, when a formal diagnosis is present, these teachers may attribute the difficulties to the diagnosis itself rather than to the child’s intentional behavior, thus reducing blame and making the relational space more accessible. In contrast, when no diagnosis is available, the same difficulties might be interpreted as signs of laziness, lack of motivation, or insufficient discipline: attributions that are likely to undermine the emotional quality of the relationship. This interpretation is consistent with previous studies showing that a clinical label can significantly recalibrate teachers’ causal attributions. Frohlich et al. (2020), for example, found that learning difficulties in students with an LD diagnosis were perceived by teachers as less stable and less controllable than those associated with ADHD, which in turn influenced their sense of self-efficacy and educational approach. Similarly, Woodcock and Vialle (2011) reported that preservice teachers were more likely to attribute poor outcomes in students with SLD to internal and uncontrollable causes, such as ability, rather than effort. Additional evidence indicates that the SLD label can activate stereotypical beliefs that alter teachers’ expectations and interpretations. Kashikar et al. (2023) demonstrated that the presence of a diagnostic label leads teachers to adopt more sympathetic and less punitive attitudes, and to frame difficulties as stable and internal, rather than as behavioral shortcomings. Likewise, Hornstra et al. (2010) observed that teachers tend to hold lower academic expectations for students with dyslexia, based on implicit beliefs associated with the label.

Taken together, these findings suggest that the presence of a diagnosis can not only influence how teachers explain difficulties, but also modulate the emotional and relational tone of their interactions with students.

Similarly, Adultcentrism showed a complex pattern: it was associated with weaker Bond with School as reported by SLD pupils, but also with higher teacher-perceived Dependency in the relationship. Notably, the latter was the only association confirmed across the entire sample, including WD pupils, suggesting that the influence of adult-centered bias may generalize beyond special needs and reflect a more general relational style. This ambivalence may point to a conception of education in which the adult plays a central, even indispensable, role – one where the teacher may feel particularly responsible framing and shaping the school experience of pupils, interpreting their closeness or reliance as signs of pedagogical success. However, as with parental blaming patterns, this strong sense of adult responsibility might reflect more the teacher’s position than the child’s actual needs or experiences.

Several authors have pointed out that the way teachers conceive of their role in the educational relationship may shape their sense of indispensability and lead to an overestimation of their own centrality. Bingham (2004) critically observed that exercising authority is a relational activity, yet educators often behave “as if authority were something they owned, something they could choose to use or not,” whereas, in fact, “authority has us rather than us having authority.” (Bingham, 2004, p. 28). This implies that the teacher’s role may be guided less by the learner’s needs and more by historically internalized logics of control and dependency. In this view, students should be “strategic enough to be against the authority of the teacher while being for the authority of the text” (Bingham, 2004, p. 36), an invitation to decenter the adult as the sole mediator of meaning and to depersonalize educational authority, allowing learners to engage with knowledge on their own terms. These reflections align with the idea that the adult’s perceived overemphasized responsibility may not always respond to the child’s actual needs, but rather to deeper, internalized cultural models of educational power.

This idea also resonates with the work of Rinaldi (2021), which is rooted in the Reggio Emilia approach, which highlights the relational nature of education as a process involving mutual influence among children, educators, and families. In this framework, roles are conceived as interdependent, and educational dialogue is not seen as a form of transmission but as a transformative encounter that resists control and predetermined outcomes, “a process of transformation where you lose absolutely the possibility of controlling the final result” (Rinaldi, 2021, p. 139), encouraging a shift away from adult-centered control and toward shared meaning-making and participatory learning.

A similar concern is raised by Maturana and Dávila (2006), who critically describe the dominant educational culture as rooted in patterns of domination, submission, and control. They argue that many educational practices reflect a deeper cultural matrix of control and hierarchical regulation, which fosters dependence and inhibits the development of autonomy.

As previously discussed, this heightened sense of adult responsibility may reflect the teacher’s perspective rather than the child’s genuine needs or experiences. Such a stance may result in an overemphasis on the adult’s regulatory function, which – while often well-intentioned – can inadvertently constrain pupils’ autonomy, emotional expression, or sense of belonging within the school context. Taken together, these findings support the idea that teachers’ educational stance profoundly shapes the quality of their relationship with pupils with SLDs (Pasta et al., 2013; Re et al., 2021; Zee et al., 2020), and they underscore the need for further research into how teachers’ beliefs and interpretative frameworks around SLD diagnoses impact inclusive practices (Cornoldi et al., 2018). These interpretations, however, should be approached with caution, as our measures of educational stance may capture only a subset of teachers’ complex belief systems. Other implicit attitudes and contextual classroom dynamics might also influence pupils’ perceptions, warranting further investigation.

### 4.1 Educational implications

The results of this explorative study highlight critical implications for educational practice and policy in contemporary inclusive schooling. As teachers face an increasingly diverse population of pupils, including those with SLDs and others whose needs are differently nuanced or not formally diagnosed, our findings underscore the misalignment of rigid and authoritarian educational frameworks. Therefore, the increase of SLD diagnoses cannot simply be addressed by adapting old-fashioned methods, which have become obsolete. Instead, our results point to the need for relationally attuned, flexible, and reflexive practices in which children are regarded as equally essential to the school and the relationship as adults. This reversal of perspective would align with the need to decenter the adult role in favor of more child-responsive approaches, challenging adultcentric assumptions that underlie Bleak Pedagogy’s authoritarian stance. It is important to underline that this view is not advocating for the erosion of the adult role, since even permissiveness—while appearing child-centered—may, as Florio et al. (2022a, 2022b) observe, reproduce adultcentric assumptions about what children want, rather than genuinely responding to their evolving agency and needs.

As also shown by the associations between teachers’ representations of SLD diagnosis and pupils’ reported Dissatisfaction, teachers are required to manage substantial changes and growing complexity. In particular, lower agreement with the perceived usefulness of diagnosis, as well as stronger endorsement of the idea that parents need more clarity regarding the roles of professionals and the nature of SLDs, predicted higher levels of Dissatisfaction among SLD pupils. These results may suggest that, in the absence of shared understanding and relational meanings surrounding the diagnosis, children may feel overlooked, as the focus shifts instead to the adult (i.e., the parent) who is expected to be trained, or may experience a lack of attunement to their specific needs—possibly because, after undergoing a labelling diagnostic process managed by adults, they are then embedded in a teacher-pupil relationship where the usefulness of that diagnosis is implicitly or explicitly questioned by the adult.

This growing complexity does not appear to be accompanied by adequate solutions or concrete support in teachers’ everyday work with children. What seems to emerge is not the need for additional top-down technical training on SLDs targeting teachers, but rather for reflective space, relational attunement, and professional recognition and evolution. Teachers need time and opportunity to adapt to fast-changing educational demands, to redefine their role in light of evolving values, and to be acknowledged for their efforts—especially when oriented toward professional growth aimed at moving beyond an authoritarian stance. This also entails the possibility to engage in equal and meaningful dialogue with parents, professionals, institutions, and potentially with children themselves. However, such engagement can only take place if teachers are given the time and manageable workloads that make it realistically feasible in everyday practice—an aspect that deserves further empirical attention. Teachers, too, have specific needs, and in the multiple relationships they maintain with pupils, a joint perspective that considers the needs of all actors becomes essential: what benefits one side may positively resonate with the other. When teachers are not supported in rethinking their role and purposes – so that they can continue evolving in response to the ever-changing demands—there is a risk that difficulties will be attributed to children’s traits and characteristics instead. This may reinforce a sense of inadequacy on both sides of the relationship. As Pianta (2001a) warned, biologically oriented explanations of school failure tend to place responsibility on children’s immutable traits – such as genetics or neurodevelopment—rather than on the quality of the relational and contextual environment that supports learning.

Our argument around authoritarian educational stances does not aim to fall into this risk: we are not referring to teachers as either possessing or lacking authoritarian traits. In line with Bingham (2004), we understand authoritarian behaviors as emergent properties of relationships—properties that, for instance, may be shaped by broader institutional condition, such as rigid school structures or accountability pressures. These systemic factors have already been explored in relation to teachers’ emotional exhaustion and burnout (Bodenheimer and Shuster, 2020; Maas et al., 2021). Given that emotional exhaustion has been shown to mediate the link between teachers’ authoritarian leadership and students’ well-being (Peng and Huang, 2024), and that under greater work pressure, teachers might find it harder to maintain the emotional attunement and investment required by students who elicit more complex support needs (Jennings and Greenberg, 2009), further investigation on how systemic factors may limit teachers’ capacity to adopt less authoritarian and more child-responsive approaches is warranted.

The moderation effect identified in our study suggests that endorsing authoritarian methods may still foster relational closeness while simultaneously reframing dependency in a more benign light. While this may appear paradoxical, it reflects a normative conception of the teacher’s role that prioritizes guidance, structure, and care over autonomy. For example, Bennett (2017) argues that students’ autonomy – conceived as a gradual achievement—should emerge progressively from an initial phase of guided self-regulation and conformity to rules. While such a perspective may appear reasonable, it illustrates a broader tendency to view autonomy as something to be earned through compliance with adult-defined order. A critical discussion of whether these principles are inherently problematic lies beyond the scope of this paper; however, in practice, they may be co-opted into educational frameworks that offer a comfortable position for adults who expect unconditional obedience, thereby limiting the possibility of challenging authoritarian attitudes. As Peng and Huang (2024) point out, students increasingly reject the notion of unchallengeable teacher authority and instead seek recognition, respect, and more egalitarian educational relationships. In a sample of university students, the authors found that higher levels of authoritarian leadership by teachers were associated with lower levels of students’ well-being, even after accounting for variables such as age, years of study, and gender. While these results concern university students, it is worth considering how similar dynamics might unfold in younger educational contexts where there are children and adolescents.

Our findings echo this tension: although Bleak Pedagogy’s values predict higher scores on Closeness as perceived by teachers in relationships with SLD pupils, they do not mitigate the Dissatisfaction and lower Closeness reported by the pupils themselves. Moreover, even the teacher’s perception of closeness may not be entirely reassuring, since our data show two aspects: first, when considering BP values in their practical form (i.e., BP’s methods), teachers themselves perceive lower levels of Closeness in the relationship with SLD pupils; second, more weekly hours spent with pupils (all, SLD and WD) predicted stronger agreement with the “Education of children over time” factor of Bleak Pedagogy. Endorsing BP’s methods emerged as a predictor of this nostalgic attitude, which was in turn increased by more weekly hours spent with pupils. This finding aligns with the notion that old-fashioned practices are implicitly viewed as more efficient and meaningful, especially today, when explicit authoritarian methods are no longer common.

This may result in considerable effort and investment toward creating an order that, however, remains misaligned with children’s actual needs, thus fueling frustration and potentially hindering the relationship. This challenging dynamic becomes even more pressing when teachers are left to reconcile outdated pedagogical frameworks with the contemporary demands of relational and inclusive professionalism.

While authoritarian nostalgia has predominantly been explored in macro-political and sociological terms – as a longing for past regimes marked by discipline, order, and centralized authority (Goidel and Goidel, 2025; Kim, 2022; Kimura et al., 2024; Webb, 2017)—we note striking parallels with teachers’ agreement with traditional representations of child-rearing, as captured by the “Education of children over time” dimension in the Bleak Pedagogy Scale. This form of authoritarian nostalgia in education, although situated in the microsystem of the classroom or family, seems to reflect a similar idealization of past values, where compliance and adult-led discipline are viewed as essential to children’s development. Such perspectives, while not explicitly political, may sustain educational stances that resist contemporary shifts toward child-responsive and inclusive practices and can negatively impact the very relational dimensions between teacher and pupil it seeks to regulate. Such an interpretation is consistent with Maturana and Dávila’s (2006) reasoning, which links educational authority to broader macrosystemic structures of social organization, suggesting that classroom dynamics based on obedience foster dependence in students and may reflect, reproduce, or be reinforced by wider cultural and institutional patterns. While our reasoning highlights intriguing parallels between authoritarian nostalgia and educational stances, their interpretation would benefit from further studies designed to capture the nuanced and evolving nature of teacher-pupil relationships, ideally combining longitudinal and qualitative approaches.

### 4.2 Study limitations and perspectives for future research

It is also important to read this study in light of its limitations. Firstly, the number of teachers and children who participated in the study limits the generalizability of the results. In fact, even though several primary schools were involved in the research, the matching process led to the exclusion of some participants’ responses from the analyses. Larger and more representative samples are needed to validate these findings and test whether the observed patterns hold across different school contexts, cultural settings, or educational systems. Multi-site studies, involving diverse geographical areas and school types, would help to reduce sampling bias and enhance generalizability. We expect that with larger and more heterogeneous samples, the observed associations between labeling, educational stance, and relationship quality would remain consistent.

Secondly, it should be noted again that STRS measurements allowed us to focus on the single pupil the teacher had in mind while answering, whereas STRQ asked children to describe their relationship with teachers in general, this limits the ability to draw strong causal inferences about dyadic relationships due to methodological asymmetry. Although this choice of instruments was consistent with our aims and considered in the interpretation of the results, future research should prioritize the use of matched teacher-student dyads. This would allow for more accurate comparisons and enable researchers to rigorously test the specificity, directionality, and potential causality of the associations observed at the group level. One hypothesis that may be tested in such future studies is whether pupils’ dissatisfaction and perceived closeness will more strongly mirror teachers’ reported levels of conflict and closeness when both report on the same relationship, particularly in the case of pupils with an SLD diagnosis. Thirdly, a limitation of our design concerns the comparison between pupils with SLD and low achievers without a diagnosis. The absence of rigorous and shared criteria for teachers to define poor academic performance restricts the extent to which the findings can be generalized when comparing pupils with an SLD diagnosis and pupils without a diagnosis in relation to academic performance. While this finer distinction allows us to disentangle potential labeling effects from performance-related factors, the low achiever group is inherently heterogeneous in terms of the underlying causes of low performance, and the evaluation of low achievement is based on teachers’ subjective judgments. Moreover, in accordance with the purposes of this work to explore the possible role that teachers’ beliefs and attitudes play in defining the quality of the pupil-teacher relationship, asking them to choose a pupil on the basis of a subjective assessment of school performance allowed us to minimize external influences on such representations. Nonetheless, future studies should establish clearer, standardized definitions of low achievement and include additional measures (e.g., objective academic performance) to better understand how specific characteristics (e.g., unrecognized learning difficulties or socio-emotional factors) may influence teacher perceptions. In particular, it may be hypothesized that teachers show more empathy and closeness toward pupils whose difficulties are perceived as structural (e.g., diagnosed cognitive impairments) compared to those perceived as related to effort or behavior, potentially revealing nuanced labeling effects beyond formal diagnosis.

Given the relatively large number of statistical tests performed, our findings should be interpreted with caution: the risk of Type I errors may be inflated, while the small sample size may have reduced statistical power, increasing the likelihood of Type II errors. Although 95% confidence intervals were reported to assess the robustness of effects, future research should replicate these results with larger samples and preregistered hypotheses to ensure reliability.

In light of the limitations and considerations discussed above, future studies could benefit from testing more comprehensive models and adopting longitudinal designs, including variables such as academic achievement and peer relationships to better understand the school experience of pupils with and without a diagnosis. It could be hypothesized, for instance, that the quality of teacher-student relationships mediates the link between pupils’ academic achievement and peer acceptance, potentially also influencing bullying dynamics, as positive teacher-student bonds may help counteract peer victimization and exclusion (Troop‐Gordon, 2015). Research should also investigate gender-based differences using designs that explore moderating effects of teacher gender or school climate, with the hypothesis that male and female pupils, with or without a diagnosis, experience less conflict in inclusive and collaborative environments. Moreover, future work could examine how teaching experience interacts with organizational well-being, workload, or emotional exhaustion, testing whether experience indirectly affects relationship quality or moderates the impact of other professional characteristics. In this regard, the marginally significant association we observed between years of teaching and Closeness with WD pupils (*r* = 0.24, *p* = 0.05) suggests that experience might exert a subtle effect on relational perceptions. Future studies could specifically test directional hypotheses about how teaching experience shapes relationship quality, using larger samples or longitudinal designs to clarify whether this reflects a meaningful trend or a context-dependent effect.

An important next step is to investigate how Adultcentrism and Bleak Pedagogy shape teacher-student relationships across different educational levels (e.g., lower and upper secondary school levels), where developmental demands and power dynamics may differ. A longitudinal design would allow testing whether high endorsement of adultcentric or authoritarian attitudes predicts a progressive reduction in students’ perceived agency and autonomy over time. Moreover, it could be hypothesized that these constructs influence not only the teacher-student bond but also the broader classroom climate, with potential effects on peer relationships, including higher risks of exclusion or bullying in contexts where hierarchical, control-oriented approaches prevail.

## 5 Conclusion

The novelty of this study lies in the systematic way in which teachers’ beliefs and attitudes towards pupils with SLD are taken into account, alongside the children’s point of view, which allows us to understand where an intervention may be needed to decrease the level of dissatisfaction in the relationship with their teachers, and at the same time to improve the quality of the teacher-student relationship in light of the new challenges arising from the decline of authoritarian stances and practices in education. These preliminary results confirm that the presence or absence of diagnosis may play a role in representations of relationship quality. In order to better understand what this role may be and which factors contribute to it, the results encourage an increasingly complex and in-depth exploration of the beliefs and attitudes that teachers, consciously or unconsciously, bring to the ongoing challenges of the current educational context, in which a strict Adultcentric view and Bleak Pedagogy prove to be counterproductive stances in building quality relationships between teachers and pupils. Further research is needed to consolidate a robust framework for targeted interventions to help teachers become more aware of their educational stance and update it in light of the fast-changing society, as they are expected to foster positive and attentive relationships with each pupil in very heterogeneous classes. The pressure to respond effectively cannot be met through the replication of old-fashioned methods. Rather than additional top-down training or rigid guidelines, our results point to the importance of providing teachers with time, space, and recognition to redefine their role, embrace uncertainty, and engage in professional transformation. This includes not only learning to support pupils with specific needs, but also critically examining their own beliefs about authority, responsibility, and the meaning of teaching.

## Data Availability

The original contributions presented in the study are included in the article/supplementary material, further inquiries can be directed to the corresponding author.
